# Supplementary material for: Macrophage-derived IL-1β/NF-κB signaling mediates parenteral nutrition-associated cholestasis
Source: Nat Commun. 2018 Apr 11;9:1393. doi: 10.1038/s41467-018-03764-1 (PMC5895696; doi:10.1038/s41467-018-03764-1)
Supplement: Supplementary file 1 — Supplementary Information [file 41467_2018_3764_MOESM1_ESM.pdf]

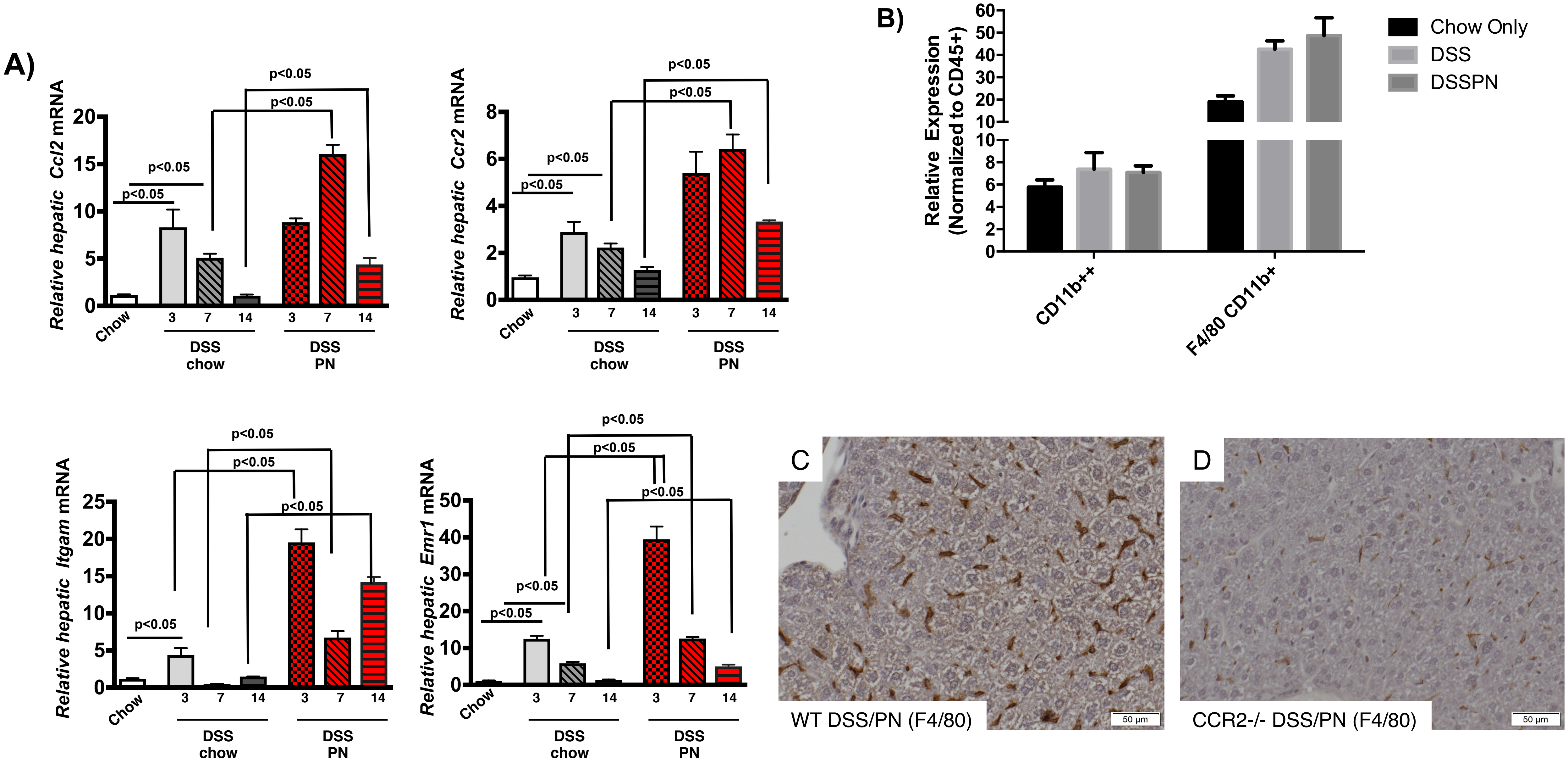

A)
Relative hepatic Ccl2 mRNA
Relative hepatic Ccr2 mRNA
Relative hepatic Itgam mRNA
Relative hepatic Emr1 mRNA
p<0.05
Chow
3
7
14
DSS chow
DSS PN
B)
Relative Expression (Normalized to CD45+)
Chow Only
DSS
DSSPN
CD11b++
F4/80 CD11b+
C
WT DSS/PN (F4/80)
50 µm
D
CCR2-/- DSS/PN (F4/80)
50 µm

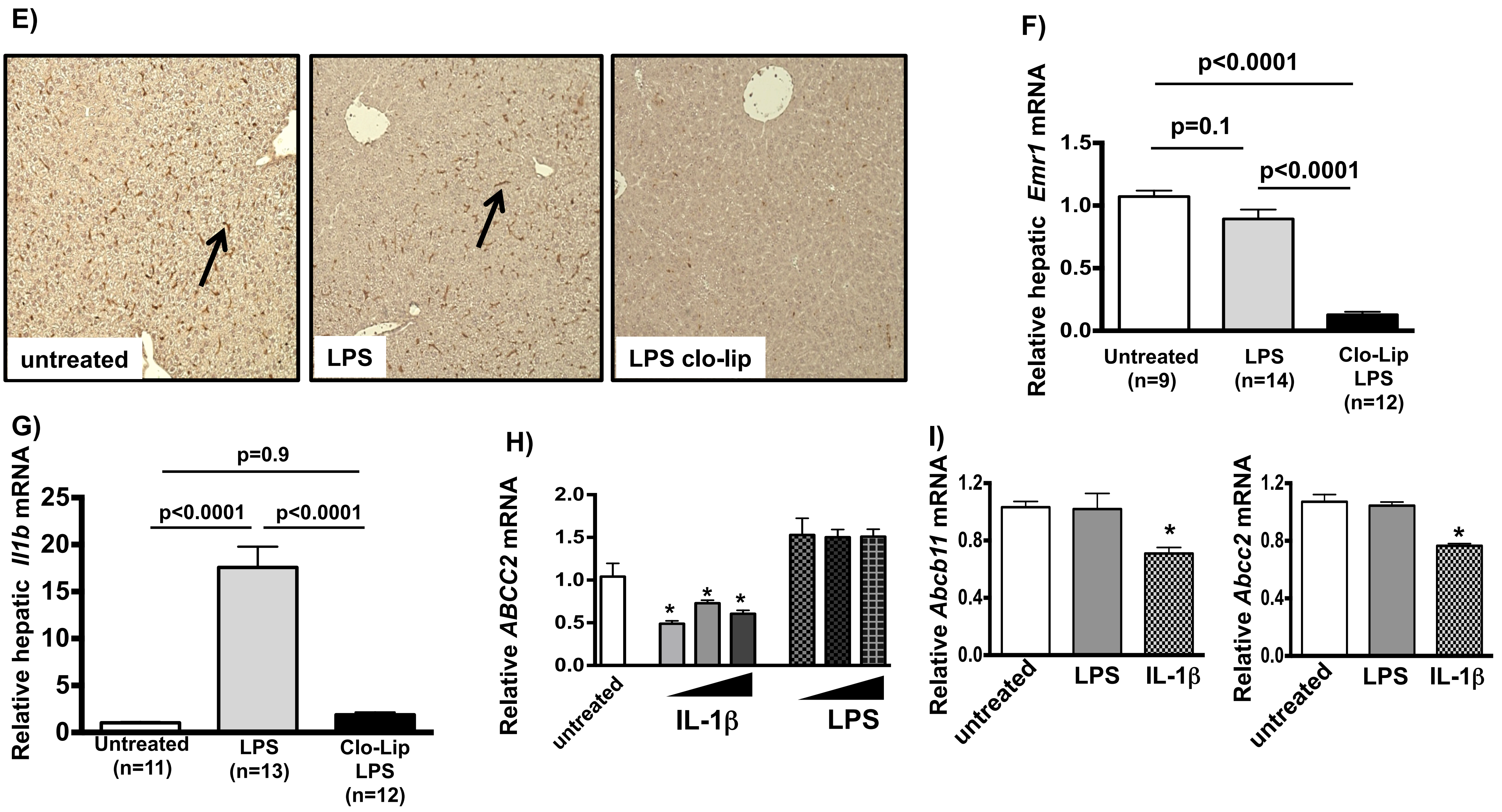
E)
untreated
LPS
LPS clo-lip
F)
p<0.0001
p=0.1
p<0.0001
Relative hepatic Emr1 mRNA
1.5
1.0
0.5
0.0
Untreated (n=9)
LPS (n=14)
Clo-Lip LPS (n=12)
G)
p=0.9
p<0.0001
p<0.0001
Relative hepatic Il1b mRNA
25
20
15
10
5
0
Untreated (n=11)
LPS (n=13)
Clo-Lip LPS (n=12)
H)
Relative ABCC2 mRNA
2.0
1.5
1.0
0.5
0.0
untreated
IL-1β
LPS
I)
Relative Abcb11 mRNA
1.2
0.8
0.4
0.0
untreated
LPS
IL-1β
Relative Abcc2 mRNA
1.2
0.8
0.4
0.0
untreated
LPS
IL-1β

J)

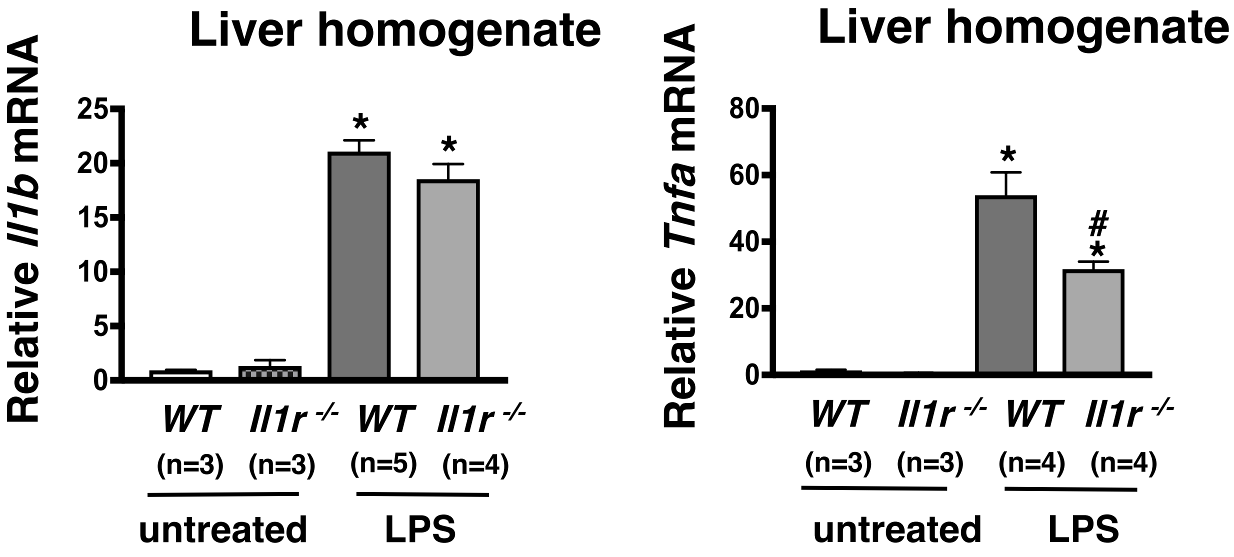
Liver homogenate
Relative Il1b mRNA
0
5
10
15
20
25
*
*
WT
Il1r -/-
WT
Il1r -/-
(n=3)
(n=3)
(n=5)
(n=4)
untreated
LPS
Liver homogenate
Relative Tnfa mRNA
0
20
40
60
80
*
#
*
WT
Il1r -/-
WT
Il1r -/-
(n=3)
(n=3)
(n=4)
(n=4)
untreated
LPS

K)

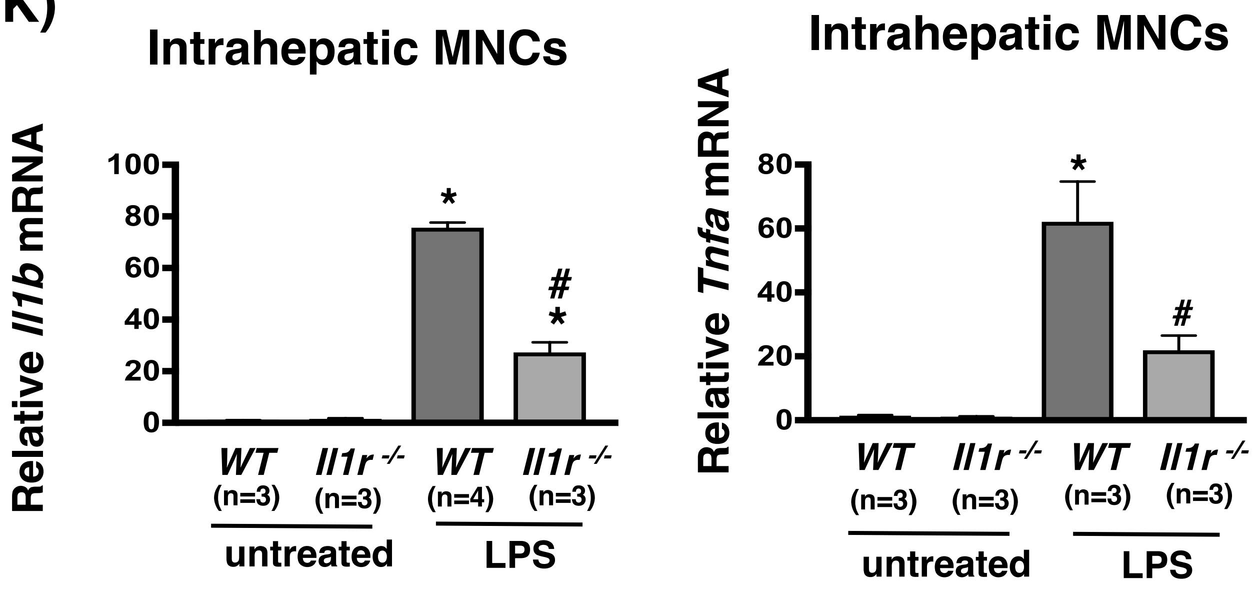
Intrahepatic MNCs
Relative Il1b mRNA
0
20
40
60
80
100
*
#
*
WT
Il1r -/-
WT
Il1r -/-
(n=3)
(n=3)
(n=4)
(n=3)
untreated
LPS
Intrahepatic MNCs
Relative Tnfa mRNA
0
20
40
60
80
*
#
WT
Il1r -/-
WT
Il1r -/-
(n=3)
(n=3)
(n=3)
(n=3)
untreated
LPS

Supplementary Figure 1

A) Hepatic gene expression (in liver homogenate) of *Ccl2*, *Ccr2*, *Itgam*, and *Emr1* depicted as relative mRNA by qPCR in DSS/chow and DSS/PN (14d time point) mice normalized to gene expression in livers of untreated chow mice. B) Gating strategy for identifying monocytes (Mono), Kupffer Cells (KCs), and recruited macrophages (recMO). Briefly, cells were selected based on FSC and SSC then singlets were selected. A Dump(-) gate was then selected which excluded neutrophils, B-cells, and T-cells. Remaining leukocytes were then selected using CD45 and segregated into mono, KCs, and recMO. Overall DSS and DSS/PN treatments increased recMO while KCs decreased modestly when compared to Chow Only controls. Relative abundance of CD11b and CD11b/F480 cells in chow, DSS, and DSs/PN livers (FACS sorted intrahepatic mononuclear cell population). C) Representative immunohistochemistry for F480 in paraffin embedded livers of WT DSS/PN mice (14d time point) and in CCR2 deficient DSS/PN mice at the same time point (D). (E) Representative immunohistochemistry for F480 in paraffin embedded livers of untreated, LPS injected (intraperitoneal 2.5mg/Kg for 4 hrs) without and with prior liposome-clodronate (LPS clo-lip) mediated macrophage ablation (24hrs prior LPS). (F, G) Hepatic gene expression (in liver homogenate) by qPCR of *Emr1* (encoding F480) and *Il1b* in the mice shown in (E); data were normalized to untreated mice. (H-I) Gene expression of *ABCC2* in Huh7 (H) in and *Abcc2* and *Abcb11* in primary mouse hepatocytes in response to LPS and IL-1b. (J,K) Gene expression of pro-inflammatory cytokines *Il1b* and *Tnfa* in liver homogenate (J) and purified intrahepatic mononuclear cells (K) of untreated and LPS treated (intraperitoneal 2.5mg/Kg for 4 hrs) WT and IL1 receptor deficient mice.

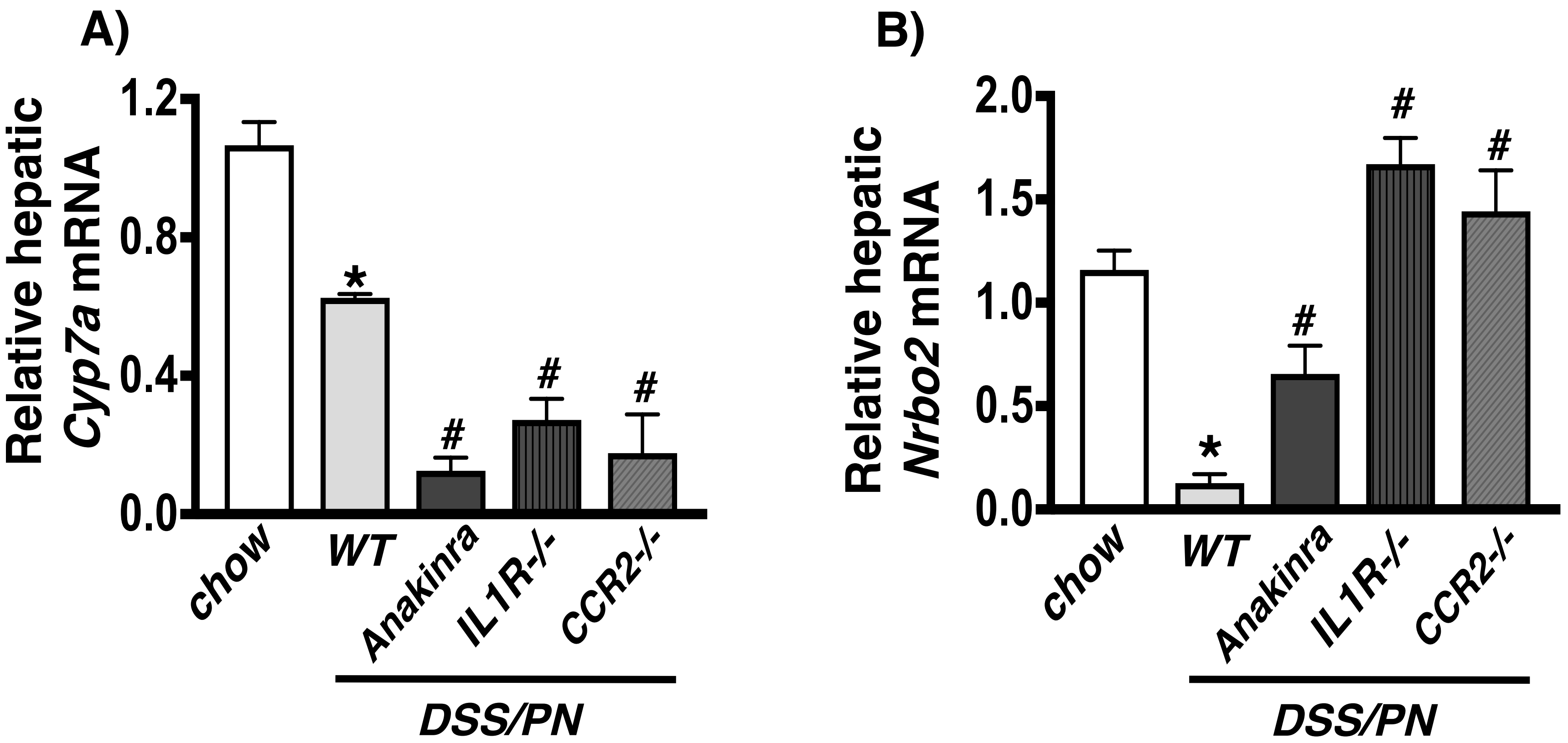

A)
Relative hepatic Cyp7a mRNA
1.2
0.8
0.4
0.0
*
#
#
#
chow
WT
Anakinra
IL1R-/-
CCR2-/-
DSS/PN
B)
Relative hepatic Nrbo2 mRNA
2.0
1.5
1.0
0.5
0.0
*
#
#
#
chow
WT
Anakinra
IL1R-/-
CCR2-/-
DSS/PN

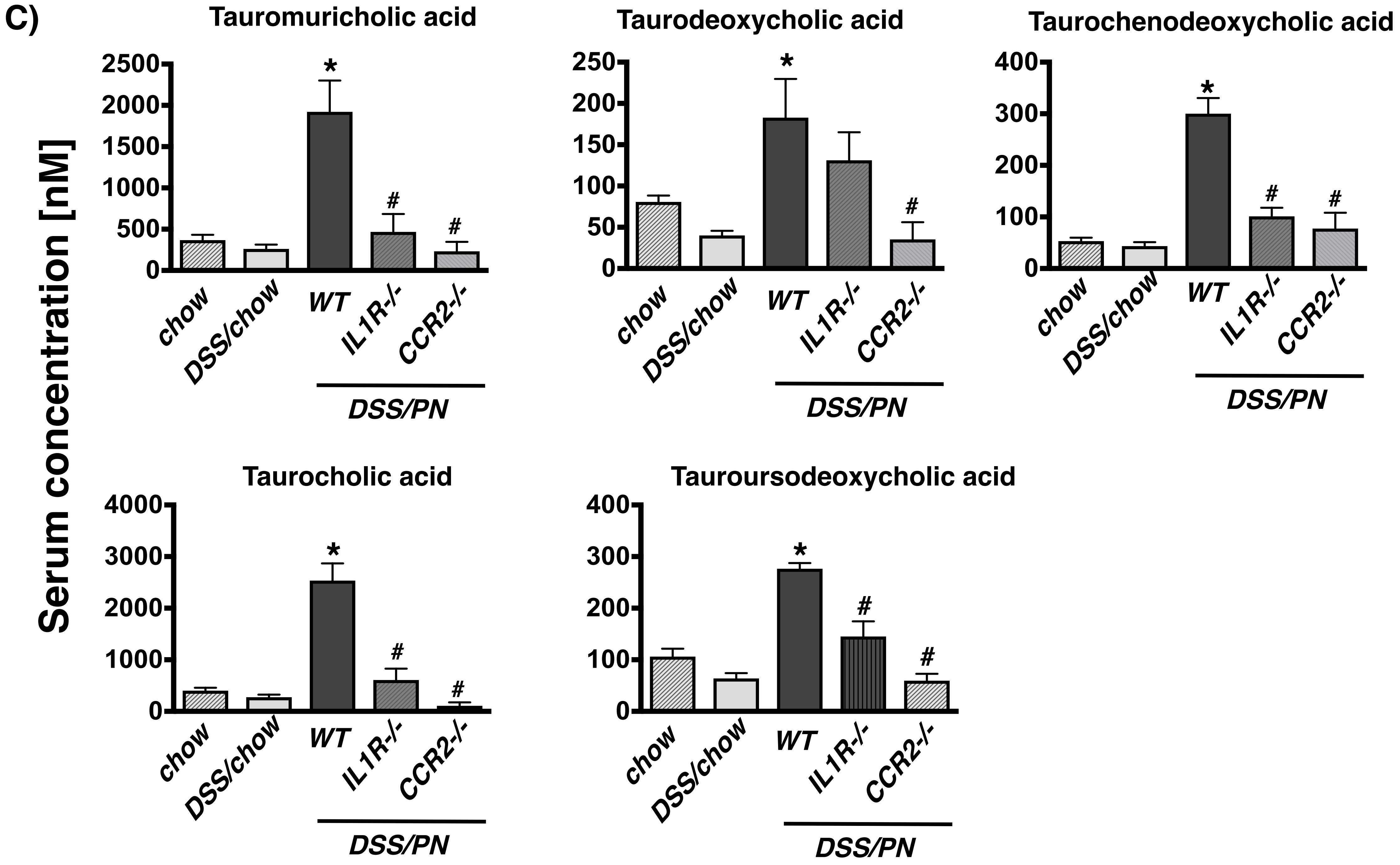

C)
Serum concentration [nM]
Tauromuricholic acid
2500
2000
1500
1000
500
0
chow
DSS/chow
WT
IL1R-/-
CCR2-/-
DSS/PN
Taurodeoxycholic acid
250
200
150
100
50
0
chow
DSS/chow
WT
IL1R-/-
CCR2-/-
DSS/PN
Taurochenodeoxycholic acid
400
300
200
100
0
chow
DSS/chow
WT
IL1R-/-
CCR2-/-
DSS/PN
Taurocholic acid
4000
3000
2000
1000
0
chow
DSS/chow
WT
IL1R-/-
CCR2-/-
DSS/PN
Tauroursodeoxycholic acid
400
300
200
100
0
chow
DSS/chow
WT
IL1R-/-
CCR2-/-
DSS/PN

Supplementary Figure 2

(A, B) Hepatic gene expression (in liver homogenate) of *Cyp7a* and *Nrbo2* depicted as relative mRNA by qPCR in wt, anakinra, IL1R ko and CCR2 ko DSS/PN mice (14d time point) normalized to gene expression in livers of untreated chow mice. (C) Serum concentrations of bile acids in wt, IL1R ko and CCR2 ko DSS/PN mice (14d time point) relative to wt DSS/chow and wt untreated mice.

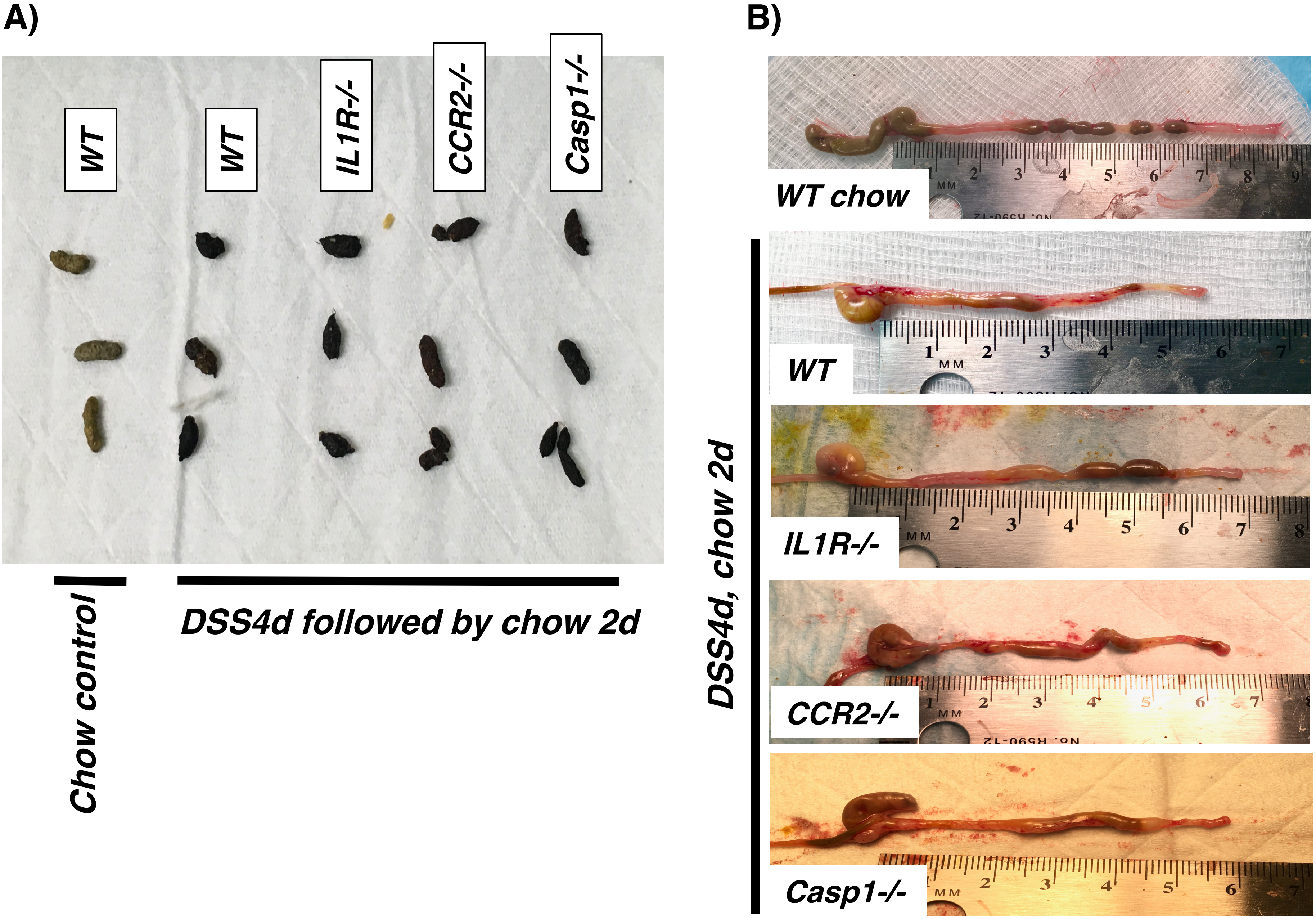
A)
WT
WT
IL1R-/-
CCR2-/-
Casp1-/-
Chow control
DSS4d followed by chow 2d
B)
WT chow
DSS4d, chow 2d
WT
IL1R-/-
CCR2-/-
Casp1-/-

C)

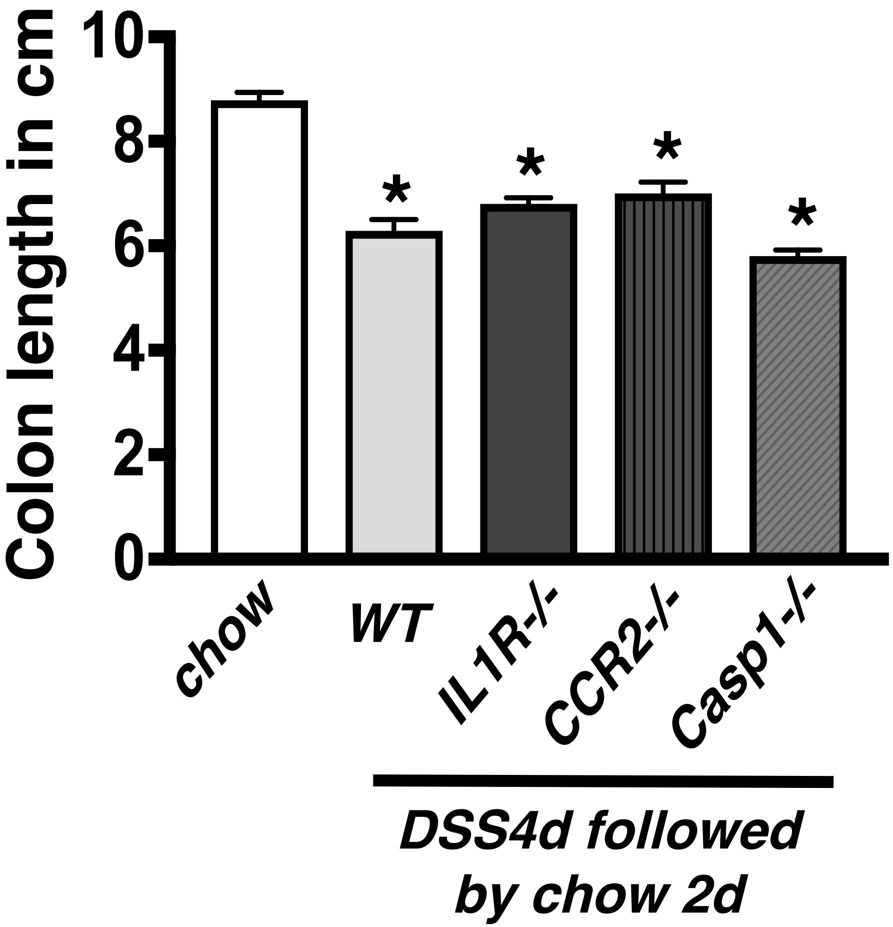

Colon length in cm
10
8
6
4
2
0
*
*
*
*
chow
WT
IL1R-/-
CCR2-/-
Casp1-/-
DSS4d followed
by chow 2d

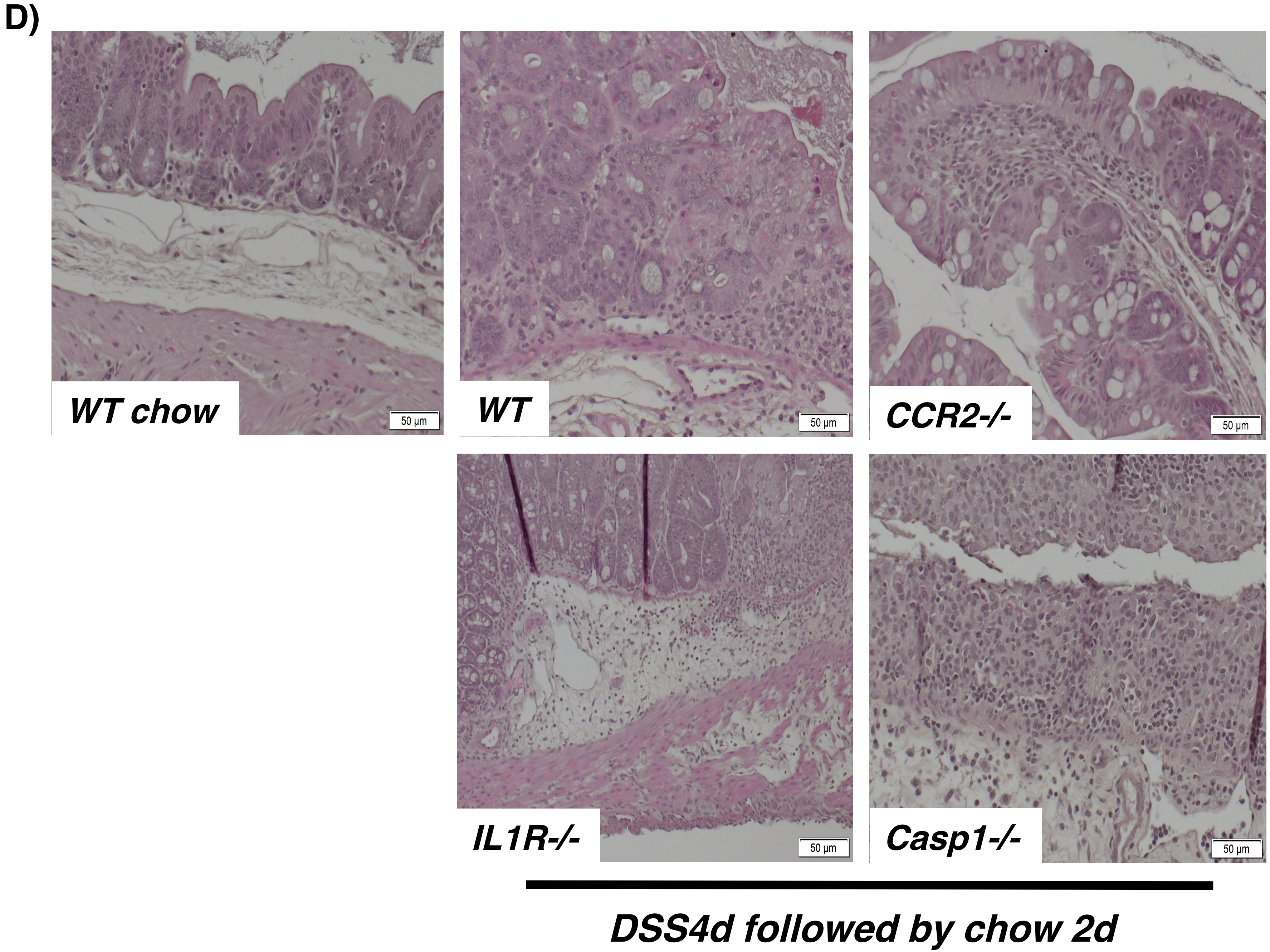
D)
WT chow
50 µm
WT
50 µm
CCR2-/-
50 µm
IL1R-/-
50 µm
Casp1-/-
50 µm
DSS4d followed by chow 2d

E)

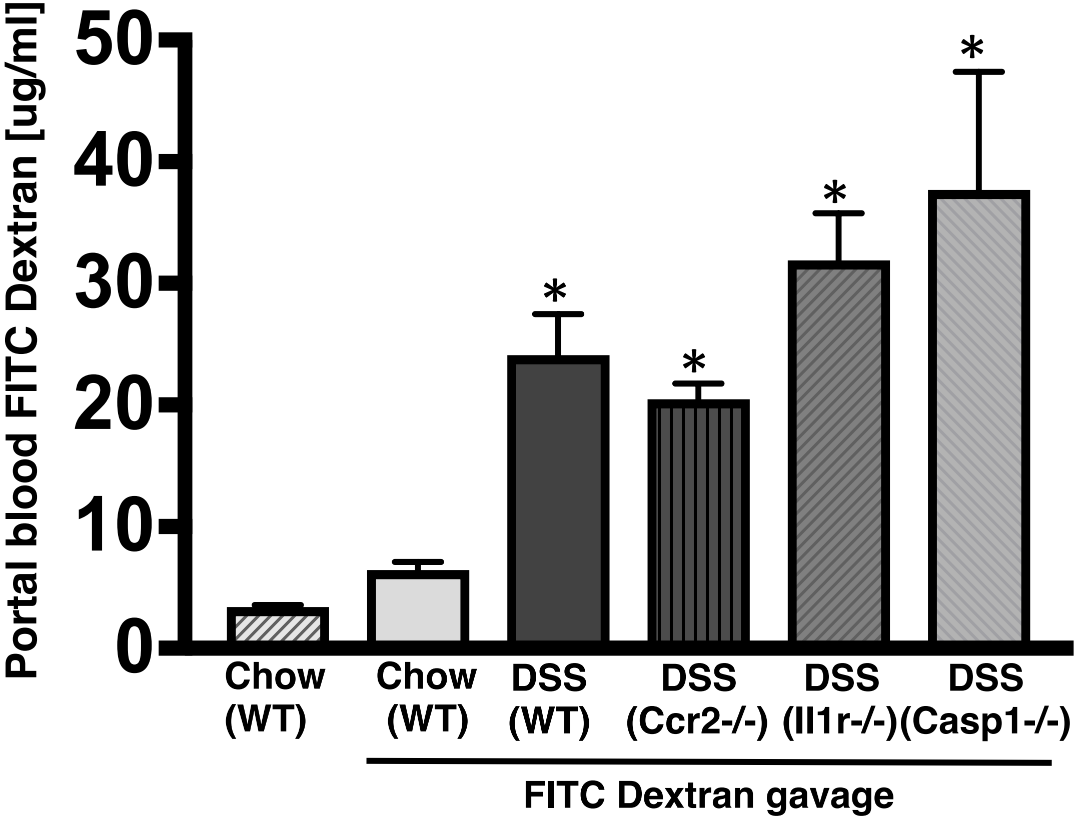

Portal blood FITC Dextran [ug/ml]
50
40
30
20
10
0
*
*
*
*
Chow (WT)
Chow (WT)
DSS (WT)
DSS (Ccr2-/-)
DSS (Il1r-/-)
DSS (Casp1-/-)
FITC Dextran gavage

Supplementary Figure 3

(A) Photographs of stool pellets collected at time of sacrifice from untreated wt chow control mice and from wt, IL1R ko, CCR2 ko, and Casp1 ko mice that had been exposed to DSS for 4 days after which DSS was discontinued for 2 additional days showing black discoloration indicative of blood in the stool pellets. (B) Representative photographs of gross colon and cecum morphology and colon length (also shown in C) in the groups of mice from (A). (D) Representative cecum histology stained with hematoxylin and eosin in the groups of mice from (A).

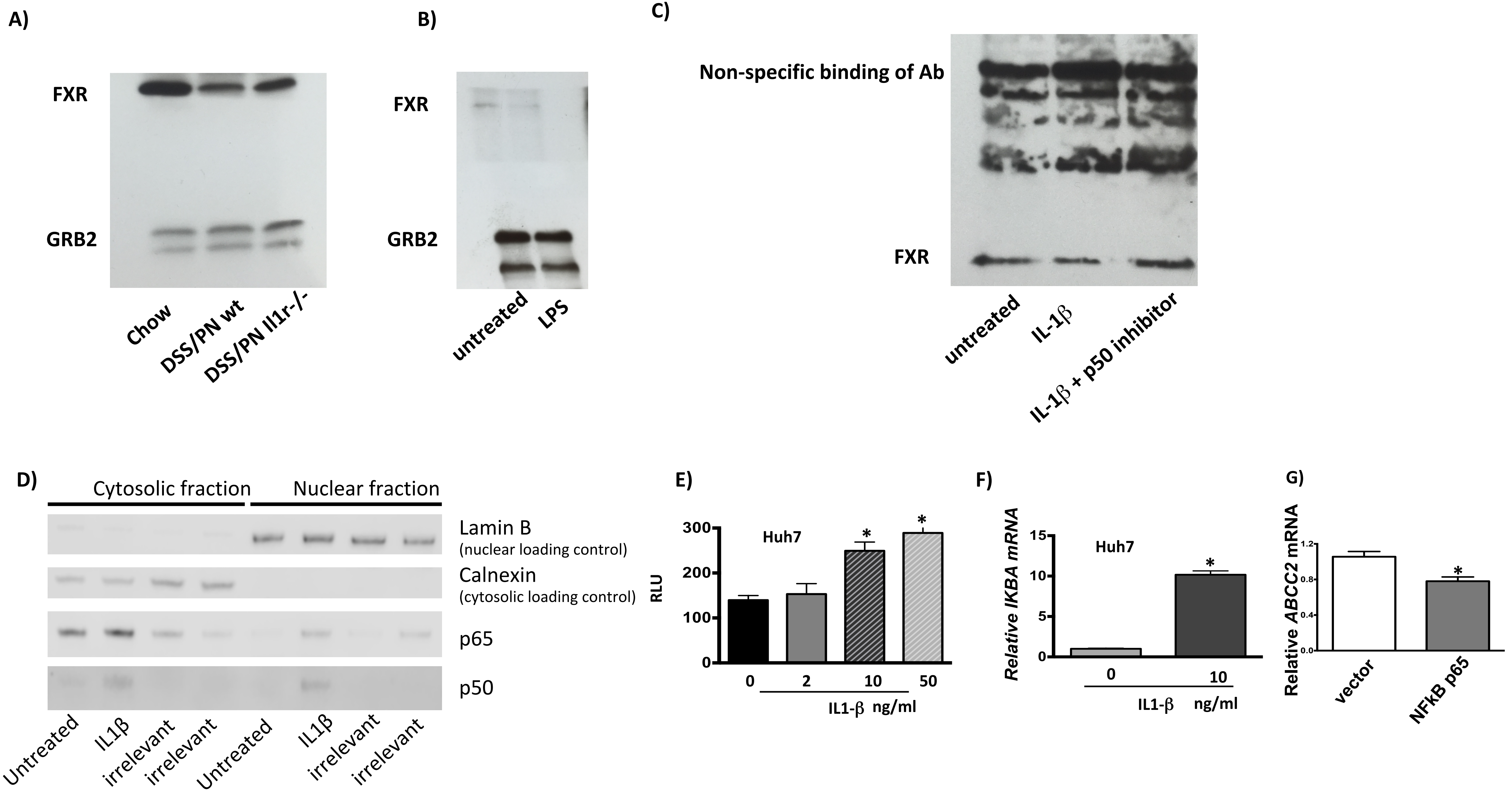
A)
FXR
GRB2
Chow
DSS/PN wt
DSS/PN Il1r-/-
B)
FXR
GRB2
untreated
LPS
C)
Non-specific binding of Ab
FXR
untreated
IL-1β
IL-1β + p50 inhibitor
D)
Cytosolic fraction
Nuclear fraction
Lamin B
(nuclear loading control)
Calnexin
(cytosolic loading control)
p65
p50
Untreated
IL1β
irrelevant
irrelevant
Untreated
IL1β
irrelevant
irrelevant
E)
Huh7
RLU
300
200
100
0
*
*
0
2
10
50
IL1-β ng/ml
F)
Huh7
Relative IKBA mRNA
15
10
5
0
*
0
10
IL1-β ng/ml
G)
Relative ABCC2 mRNA
1.2
0.8
0.4
0.0
*
vector
NFkB p65

Supplementary Figure 4

(A, B) Western blot for FXR in liver homogenate from chow mice, WT and IL1R ko DSS/PN mice (14d time point) or untreated mice and LPS injected mice (intraperitoneal 2.5mg/Kg for 4 hrs). (C) Western blot for FXR in Huh7 cells left untreated or exposed to recombinant IL1b (4hrs) without or with prior exposure to NFkb p50 inhibitor (1hr pretreatement). (D) Western blot for nuclear NFkB subunits p50 and p65 in untreated or IL1b treated Huh7 cells. (E) Luciferase activity expressed as relative light units (RLU) in IL1b treated (4hrs) Huh7 cells transfected with NFkB reporter plasmid. (F) Relative mRNA of NFkB target gene IKBA in IL1b exposed (4hrs) Huh7 cells. (G) Relative mRNA of ABCC2 in Huh7 cells transfected with an NFkB p65 overexpression plasmid (48hrs).

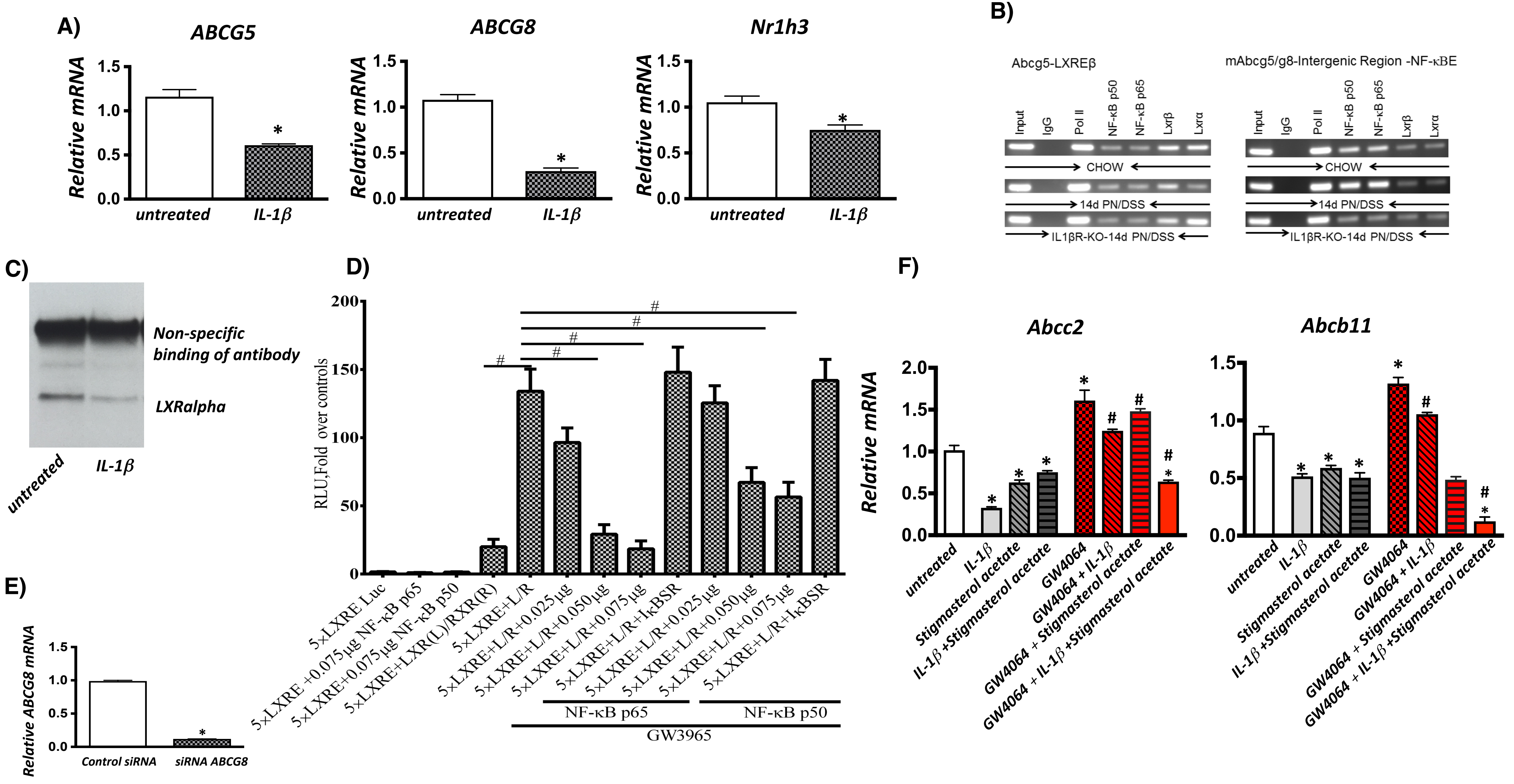
A)
ABCG5
ABCG8
Nr1h3
Relative mRNA
untreated
IL-1β
B)
Abcg5-LXREβ
mAbcg5/g8-Intergenic Region -NF-κBE
Input
IgG
Pol II
NF-κB p50
NF-κB p65
Lxrβ
Lxrα
CHOW
14d PN/DSS
IL1βR-KO-14d PN/DSS
C)
Non-specific binding of antibody
LXRalpha
untreated
IL-1β
D)
RLU,Fold over controls
5×LXRE Luc
5×LXRE +0.075µg NF-κB p65
5×LXRE+0.075µg NF-κB p50
5×LXRE+LXR(L)/RXR(R)
5×LXRE+L/R
5×LXRE+L/R+0.025µg
5×LXRE+L/R+0.050µg
5×LXRE+L/R+0.075µg
5×LXRE+L/R+IκBSR
5×LXRE+L/R+0.025µg
5×LXRE+L/R+0.050µg
5×LXRE+L/R+0.075µg
5×LXRE+L/R+IκBSR
NF-κB p65
NF-κB p50
GW3965
E)
Relative ABCG8 mRNA
Control siRNA
siRNA ABCG8
F)
Abcc2
Abcb11
Relative mRNA
untreated
IL-1β
Stigmasterol acetate
IL-1β +Stigmasterol acetate
GW4064
GW4064 + IL-1β
GW4064 + Stigmasterol acetate
GW4064 + IL-1β +Stigmasterol acetate

Supplementary Figure 5

(A) Relative mRNA of ABCG5, ABCG8, and NR1H3 in Huh7 cells left untreated or exposed to IL1b (10ng/ml for 4hrs). (B) ChIP in liver homogenate from untreated chow, WT and IL1R ko DSS/PN mice (14d time point) for binding of LXR and NFkB to the ABCG5 promoter. Left hand panel shows increased LXR alpha and beta binding to the LXRbeta response element in the ABCG5 promoter region (ABCG5-LXREb) in IL1R ko DSS/PN livers relative to WT DSS/PN livers. Right hand panel shoes binding of NFkB p50 and p65 to the intergenic region of the Abcg5/8 promoter, which was increased in WT DSS/PN livers relative IL1R ko livers. (C) Western blot for LXRalpha in untreated and IL1b exposed (10ng/ml, 4hrs) Huh7 cells. (D) Luciferase activity measured in relative light units (RLU) shown as fold over untreated controls after transfection of HepG2 cells with luciferase reporter plasmids (Luc) containing five consecutive LXR response elements (5xLXRE) with or without concomitant overexpression of LXR (L) and RXR (R) and varying concentrations of transfection with NFkB p65 and p50 plasmids with and without presence of expression of NFkB super repressor (IKBSR) in the presence of an LXR agonist (GW3965). (E) Relative mRNA for ABCG8 after 48hrs after transfection with siRNA for ABCG8 in HepG2 cells. (F) Relative mRNA of Abcc2 or Abcb11 in primary hepatocytes exposed to combinations of Il1b, stigmasterol and FXR agonist GW4064.

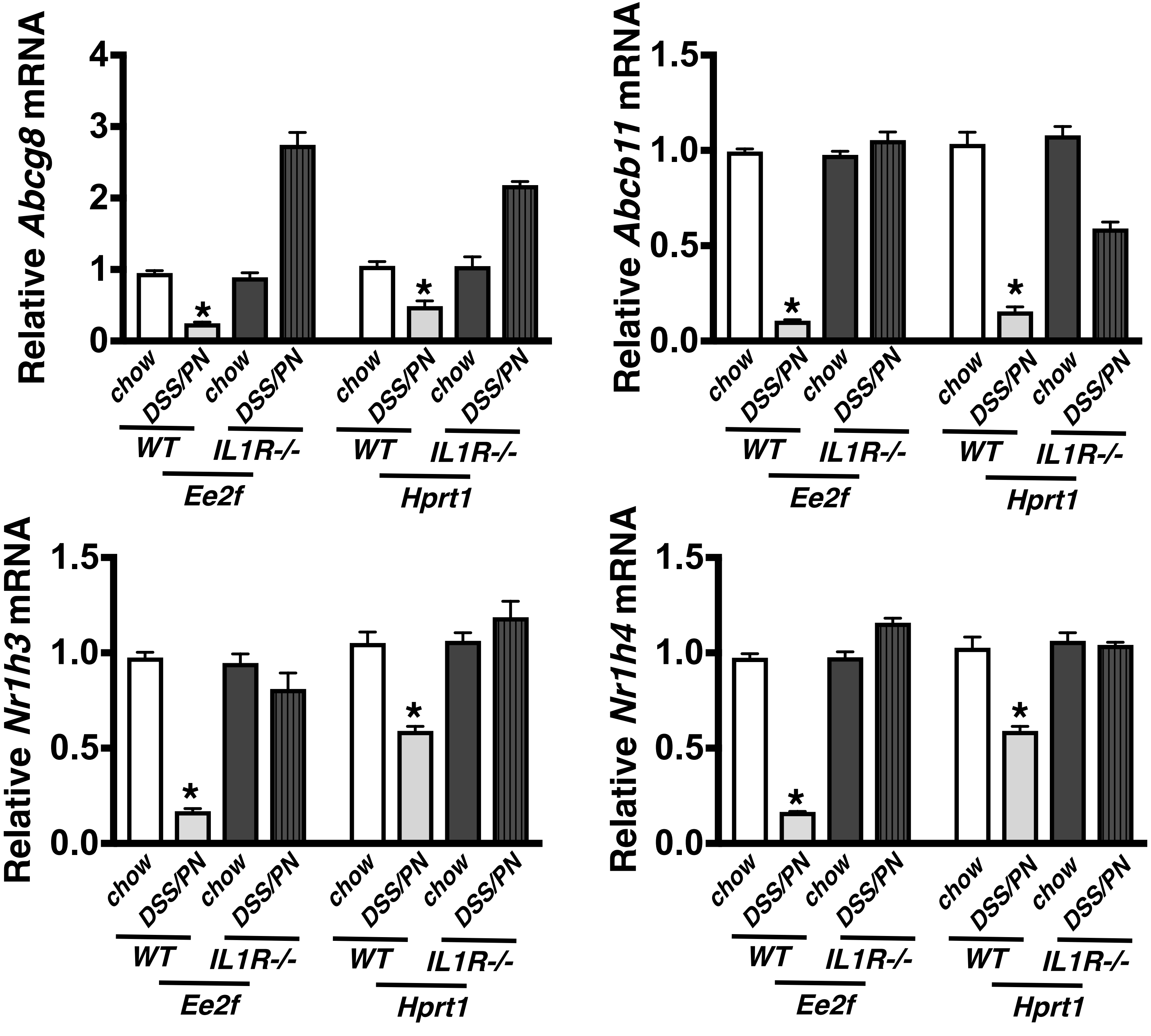

Relative Abcg8 mRNA
4
3
2
1
0
chow
DSS/PN
chow
DSS/PN
chow
DSS/PN
chow
DSS/PN
WT
IL1R-/-
WT
IL1R-/-
Ee2f
Hprt1
Relative Abcb11 mRNA
1.5
1.0
0.5
0.0
Relative Nr1h3 mRNA
Relative Nr1h4 mRNA
*

Supplementary Figure 6:

Relative hepatic mRNA of *Abcg8, Abcb11, Nr1h3,* and *Nr1h4* in liver homogenate of groups of mice (n=3-5) either WT or IL1R ko and either untreated (chow) or DSS/PN treated (14d time point) with either *Ee2f* or *Hprt1* as a house keeping gene.

A

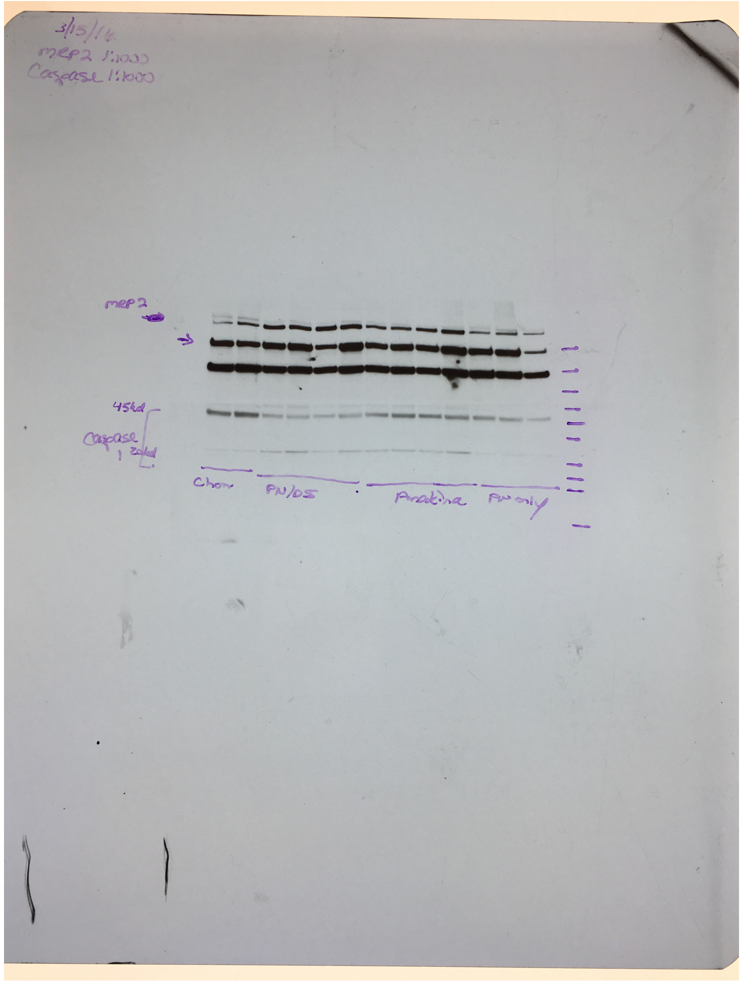

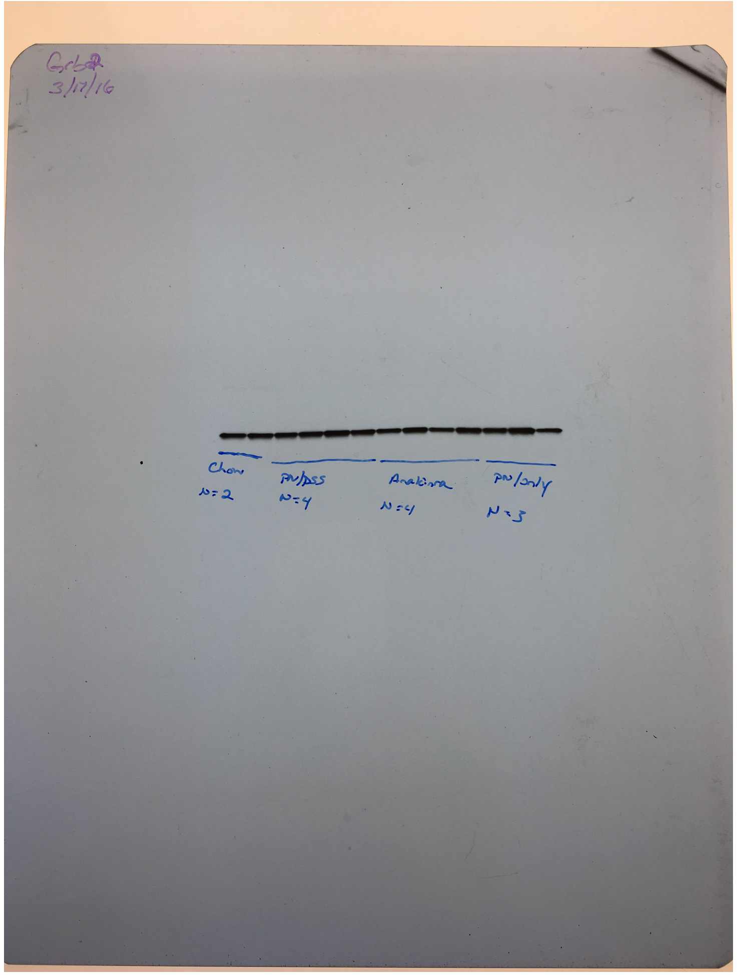

Scans of Western images shown in Figure 2F.

B

Figure 4G1-1

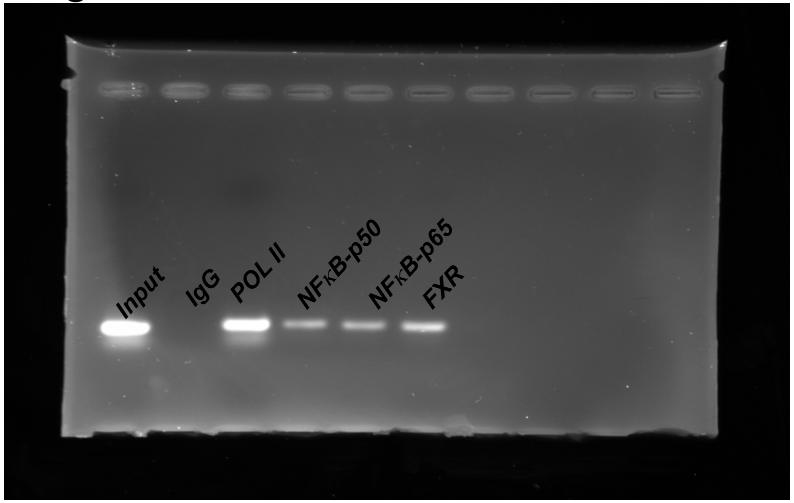

Chow

Figure 4G1-2

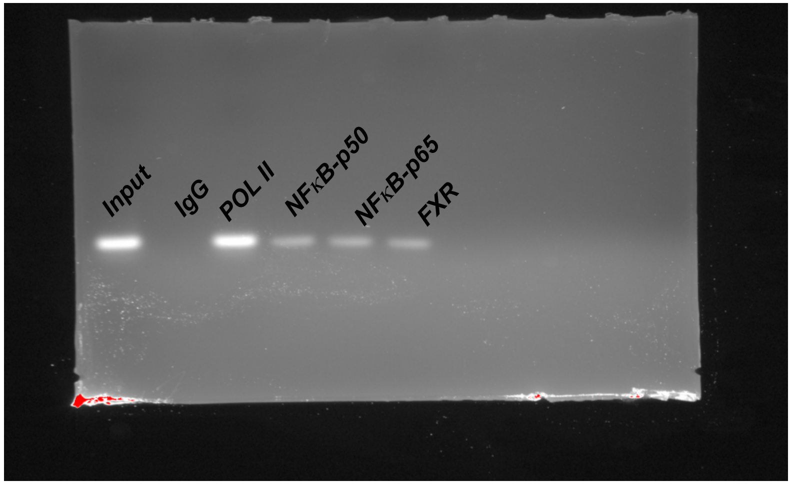

DSS/PN wt

Figure 4G1-3

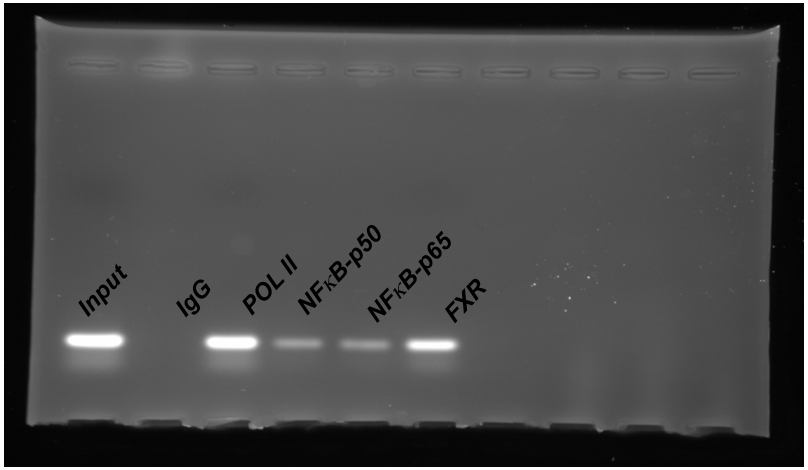

DSS/PN Il1r $^{-/-}$

Showing full gels for Figure 4 and 5 bmBsep Promoter FXRE

Chromatin immuno precipitation (ChIP) on liver homogenate from WT chow mice, WT DSS/PN mice, and *Il1r$^{-/-}$*DSS/PN mice probing with specific antibodies for either FXR , NFκB p50 and p65 subunits binding to the mouse Abcb11 promotor to FXRE binding site. Represented as semi-quantitative data.

mBsep promoter NFκBE

Figure 4G2-1

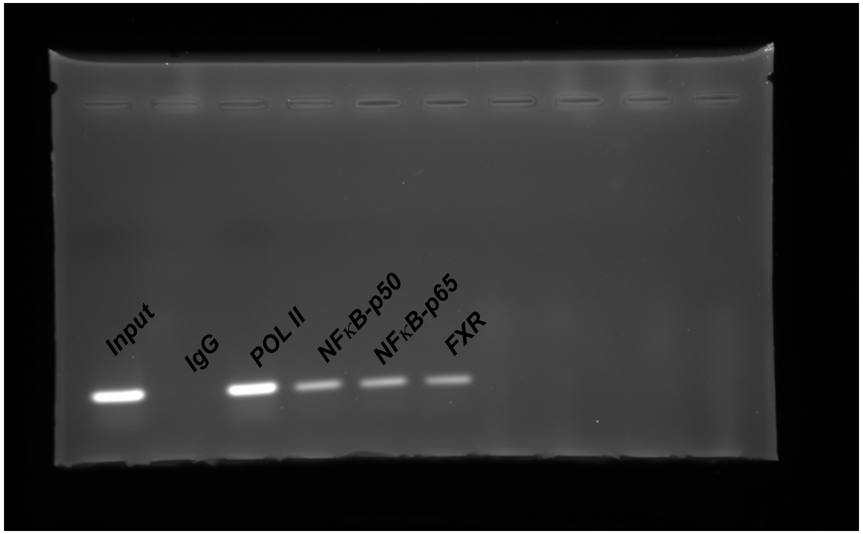

Chow

Figure 4G2-2

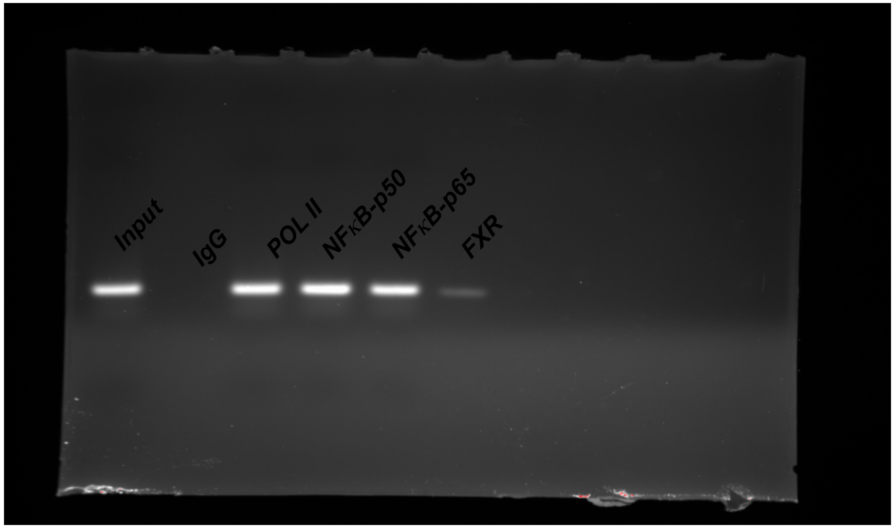

DSS/PN wt

Figure 4G2-3

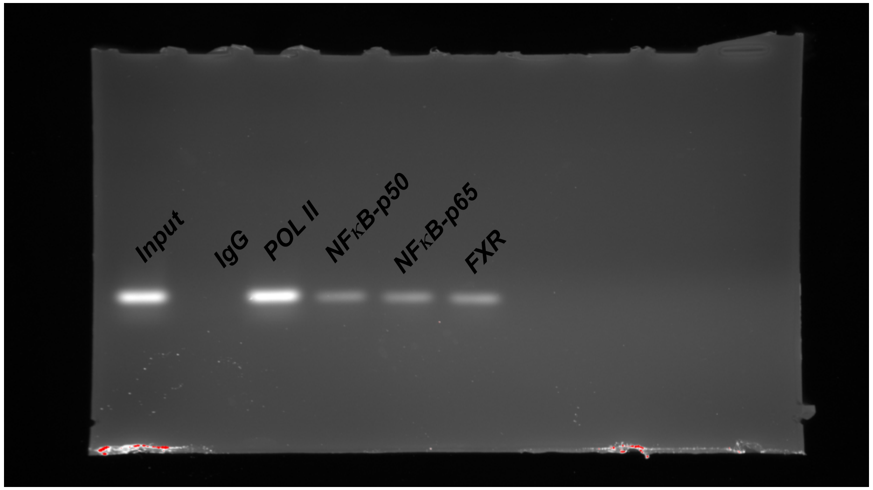

DSS/PN Il1r $^{-/-}$

Chromatin immuno precipitation (ChIP) on liver homogenate from WT chow mice, WT DSS/PN mice, and *Il1r*$^{-/-}$DSS/PN mice probing with specific antibodies for either FXR , NFκB p50 and p65 subunits binding to the mouse Abcb11 promotor to NFκBE binding site. Represented as semi-quantitative data.

mFxr promoter FXRE

Figure 4H1-1

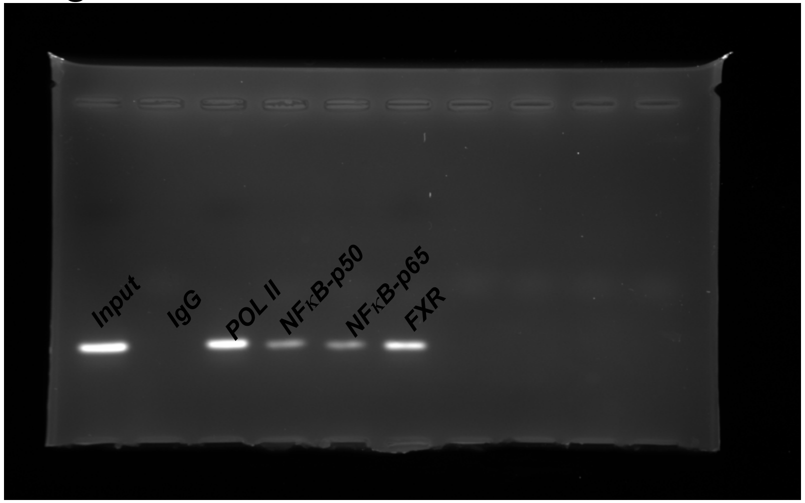

Chow

Figure 4H1-2

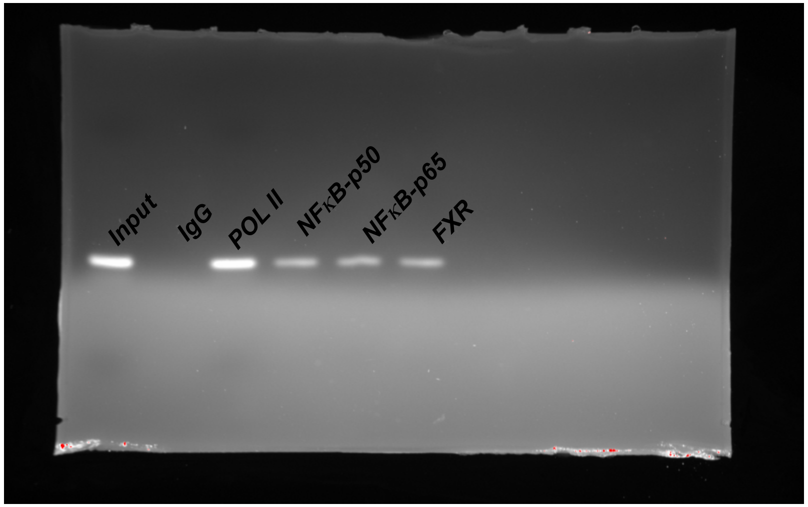

DSS/PN wt

Figure 4H1-3

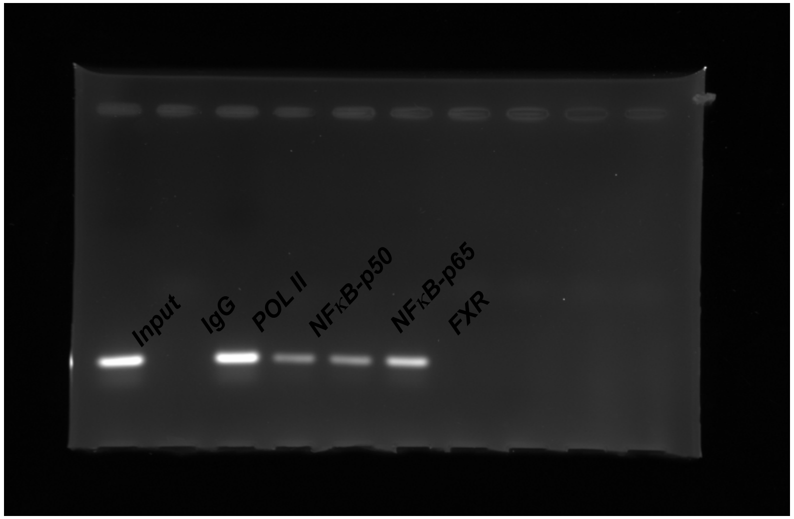

DSS/PN Il1r $^{-/-}$

Chromatin immuno precipitation (ChIP) on liver homogenate from WT chow mice, WT DSS/PN mice, and *Il1r*$^{-/-}$DSS/PN mice probing with specific antibodies for either FXR , NFκB p50 and p65 subunits binding to the mouse Nr1h4(Fxr) promotor to FXRE binding site. Represented as semi-quantitative data.

mFxr promoter NFκBE

Figure 4H2-1

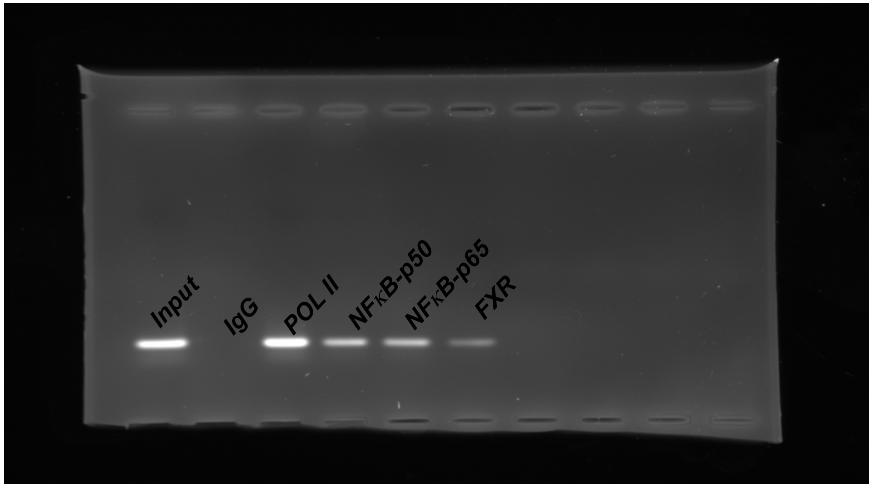

Chow

Figure 4H2-2

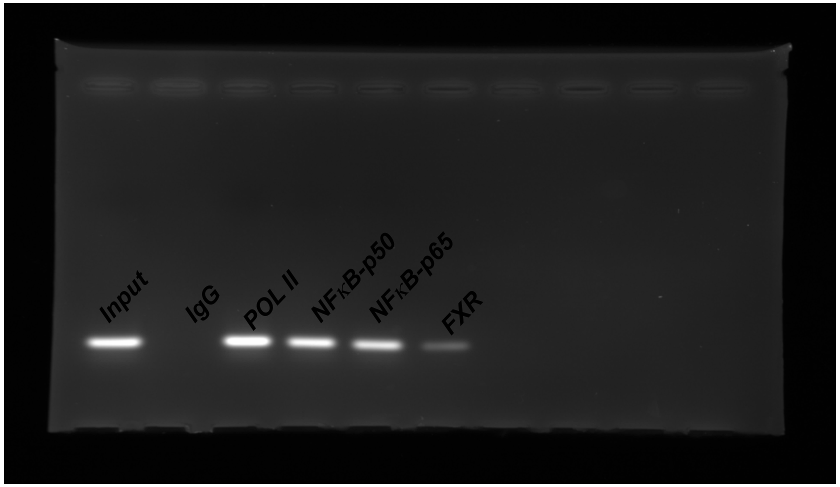

DSS/PN wt

Figure 4H2-3

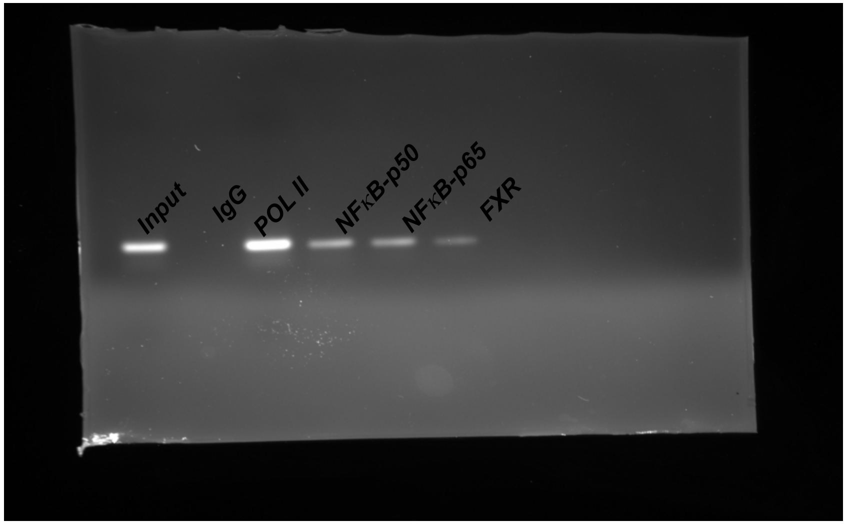

DSS/PN Il1r $^{-/-}$

Chromatin immuno precipitation (ChIP) on liver homogenate from WT chow mice, WT DSS/PN mice, and *Il1r*$^{-/-}$DSS/PN mice probing with specific antibodies for either FXR , NFκB p50 and p65 subunits binding to the mouse Nr1h4(Fxr) promotor to NFκBE binding site. Represented as semi-quantitative data.

LXR promoter LXRE

Figure5 F1-1

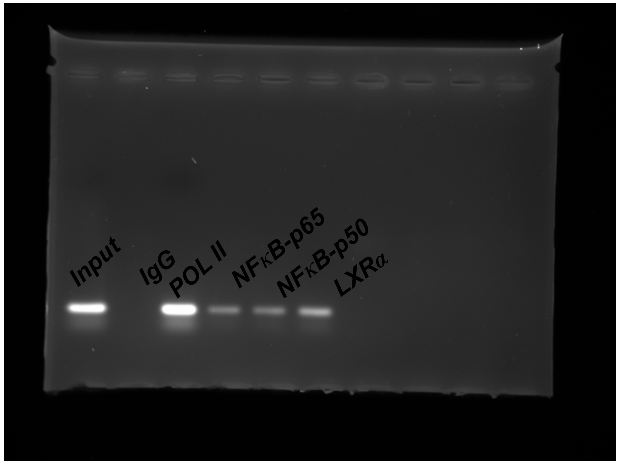

Huh7

Figure5 F1-2

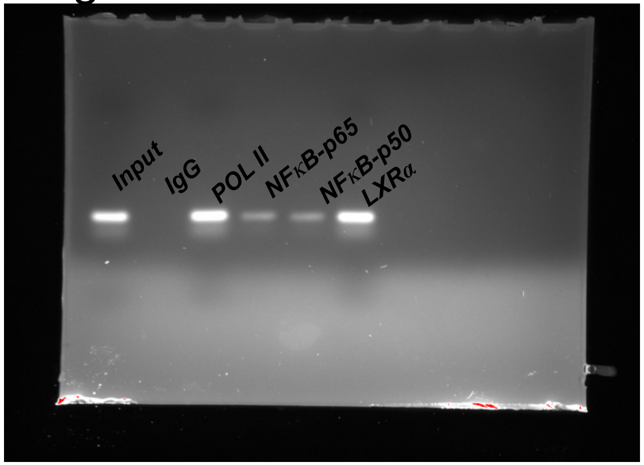

Huh7+GW3965

Figure5 F1-3

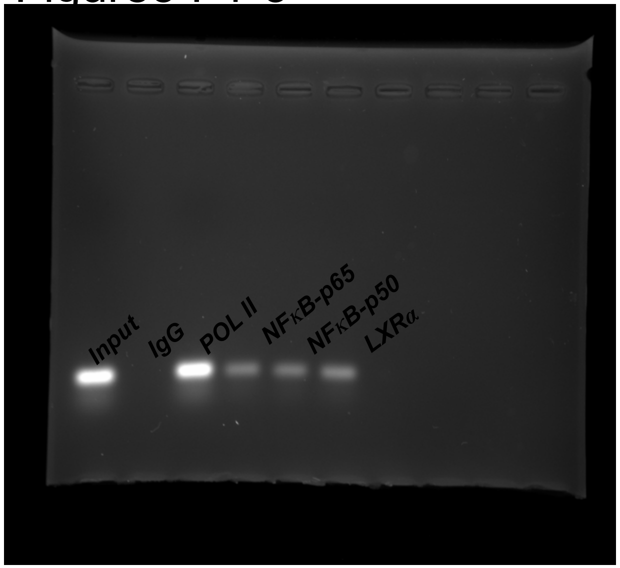

Huh7+IL-1β

Figure5 F1-4

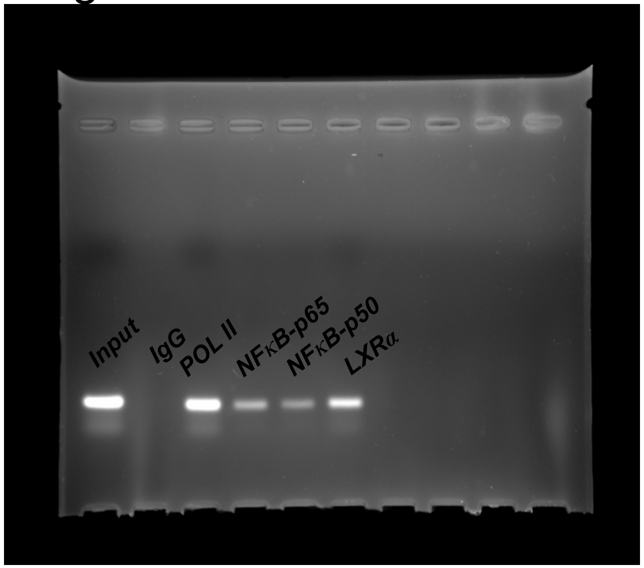

Huh7+GW3965+IL-1β

ChIP from HuH7 cells untreated, or exposed to LXR agonist (GW3965,2mM;HuH7 + GW3965) for 16 hrs with (HuH7 + GW3965 + IL-1β) or without (HuH7 + IL-1β) additional exposure to IL-1β for4hrs.Specific antibodies for either LXR , NFκB p50 and p65 subunits binding to the human NRIH3(LXR) promotor to LXRE binding site. Represented as semi-quantitative data.

LXR promoter NFκBE

Figure5F 2-1

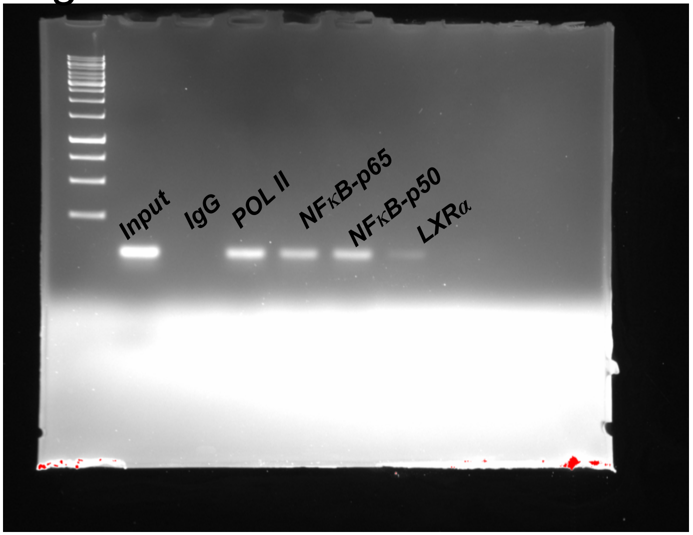

Huh7

Figure5F 2-2

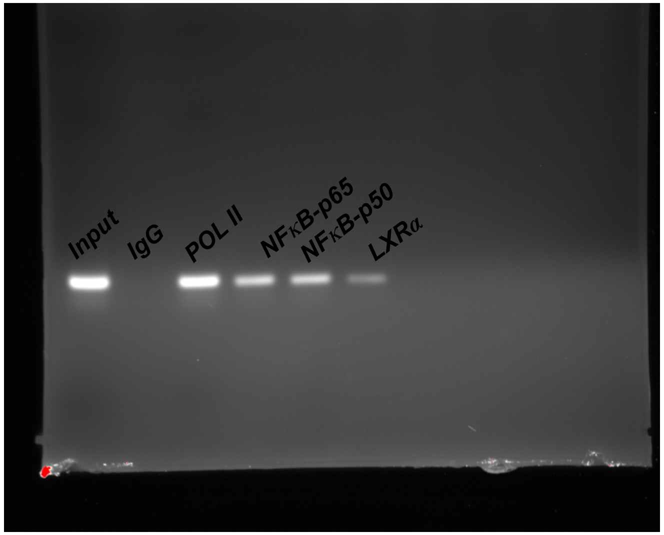

Huh7+GW3965

ChIP from HuH7 cells untreated, or exposed to LXR agonist (GW3965,2mM;HuH7 + GW3965) for 16 hrs with (HuH7 + GW3965 + IL-1β) or without (HuH7 + IL-1β) additional exposure to IL-1β for4hrs.Specific antibodies for either LXR ,NFκB p50 and p65 subunits binding to the humanNRIH3(LXR) promotor to NFκBE binding site. Represented as semi-quantitative data.

Figure5F 2-3

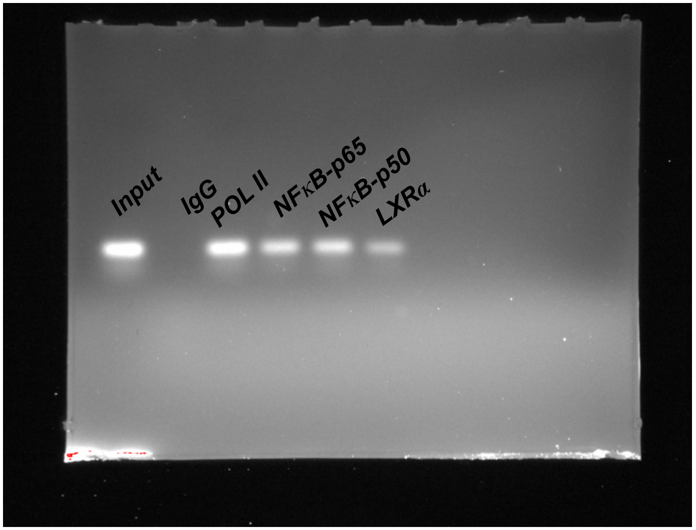

Huh7+IL-1β

Figure5F 2-4

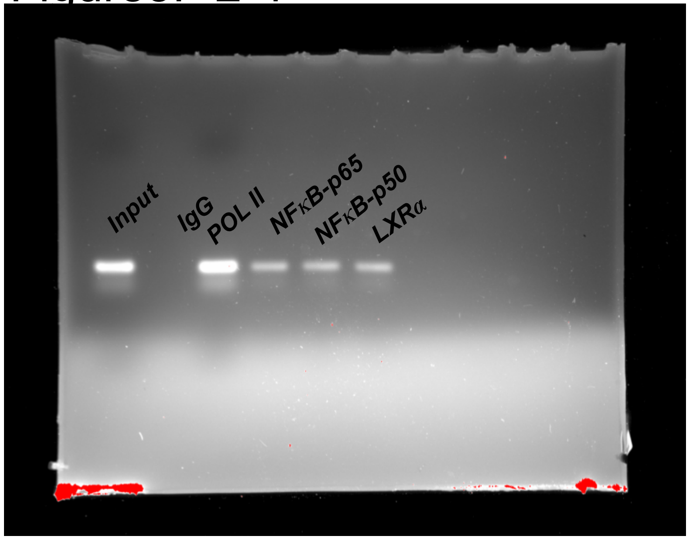

Huh7+GW3965+IL-1β

Abcg5/8 LXRβE

Figure5F 3-1

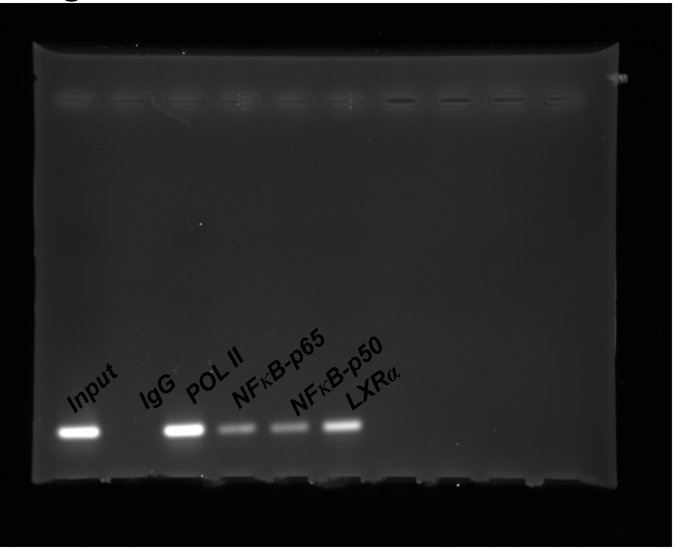

Huh7

Figure5F 3-2

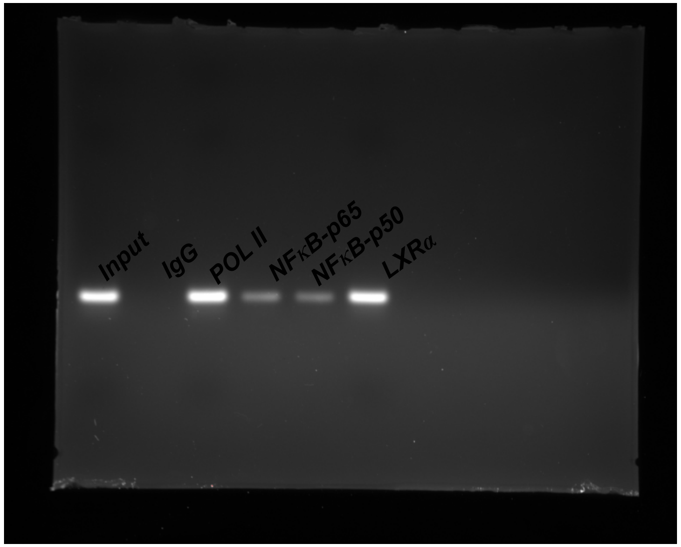

Huh7+GW3965

ChIP from HuH7 cells untreated, or exposed to LXRagonist(GW3965,2mM;HuH7 + GW3965) for 16 hrs with (HuH7 + GW3965 + IL-1β) or without (HuH7 + IL-1β) additional exposure to IL-1β for 4hrs.Specific antibodies for either LXRα , NFκB p50 and p65 subunits binding to the human ABCG5/8 promoter to LXRβE binding site. .Represented as semi-quantitative data.

Figure5F 3-3

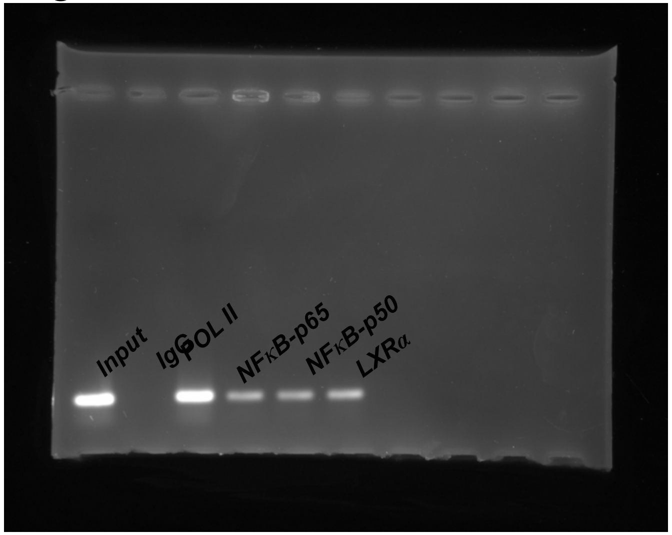

Huh7+IL-1β

Figure5F 3-4

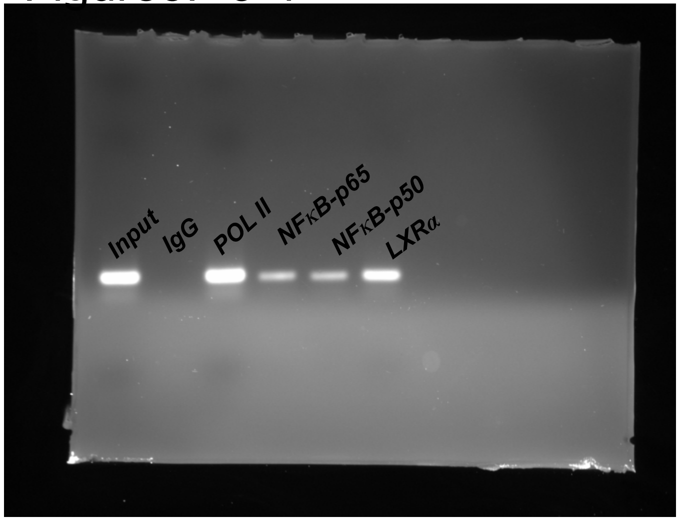

Huh7+GW3965+IL-1β

Abcg5/8 NFκBE (intergenic region)

Figure5F 4-1

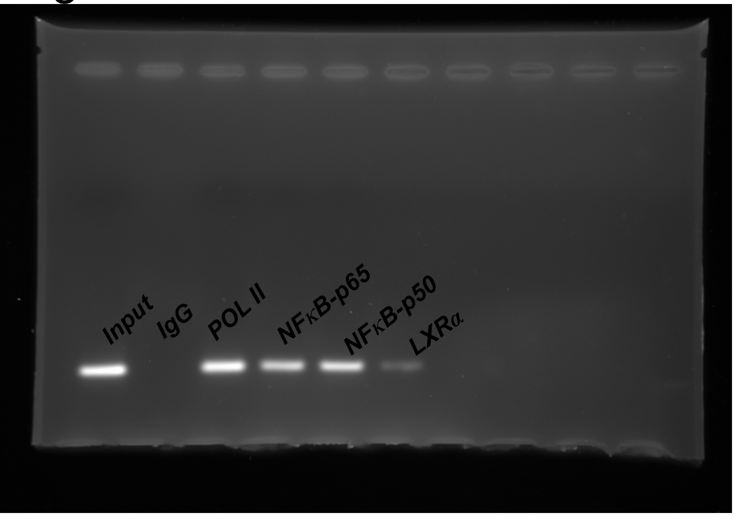

Huh7

Figure5F 4-2

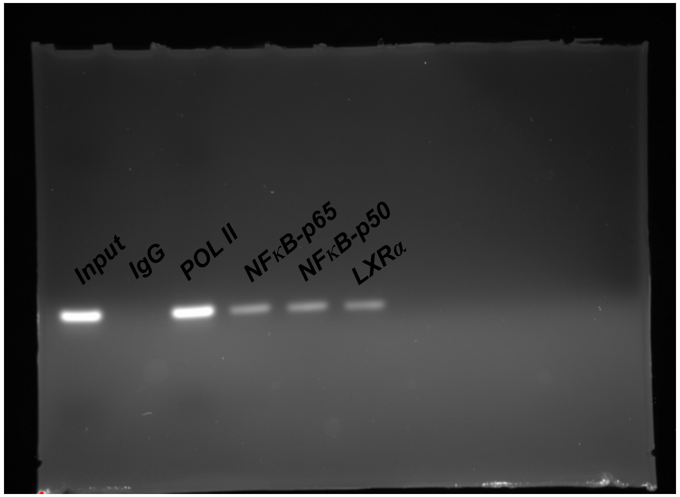

Huh7+GW3965

Figure5F4-3

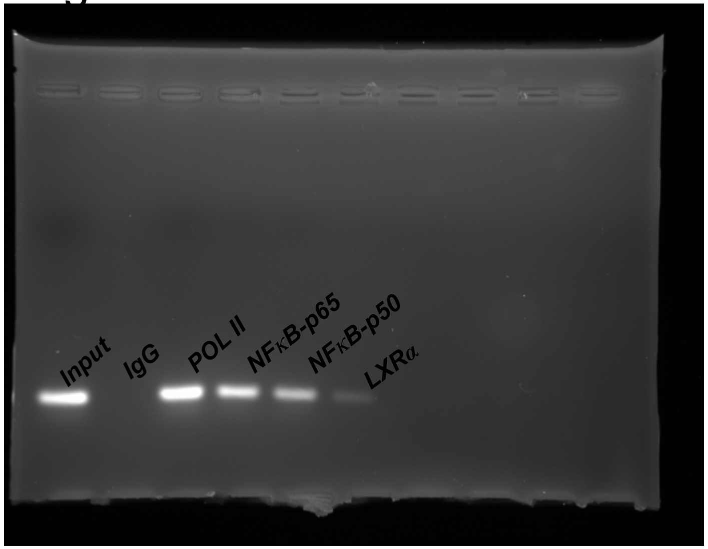

Huh7+IL-1β

Figure5F4-4

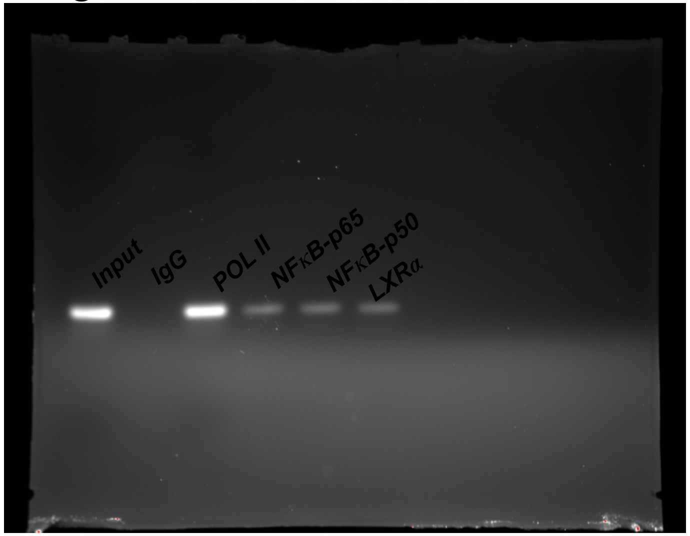

Huh7+GW3965+IL-1β

ChIP from HuH7 cells untreated,or exposed to LXRagonist(GW3965,2mM; HuH7+GW3965) for 16 hrs with (HuH7 + GW3965 + IL-1β) or without (HuH7 + IL-1β) additional exposure to IL-1β for 4hrs.Specific antibodies for either LXRα , NFκB p50 and p65 subunits binding to the humanABCG5/8 (intergenic region) promoter to NFκBE binding site.Represented as semi-quantitative data.

Abcg5-LXRβE

Supplementary Figure5B-1-1

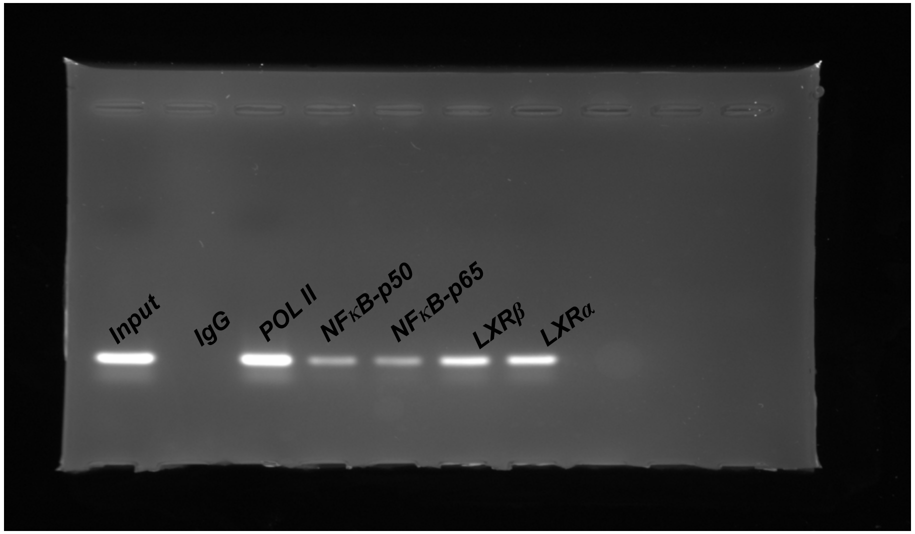

CHOW

Supplementary Figure5B-1-2

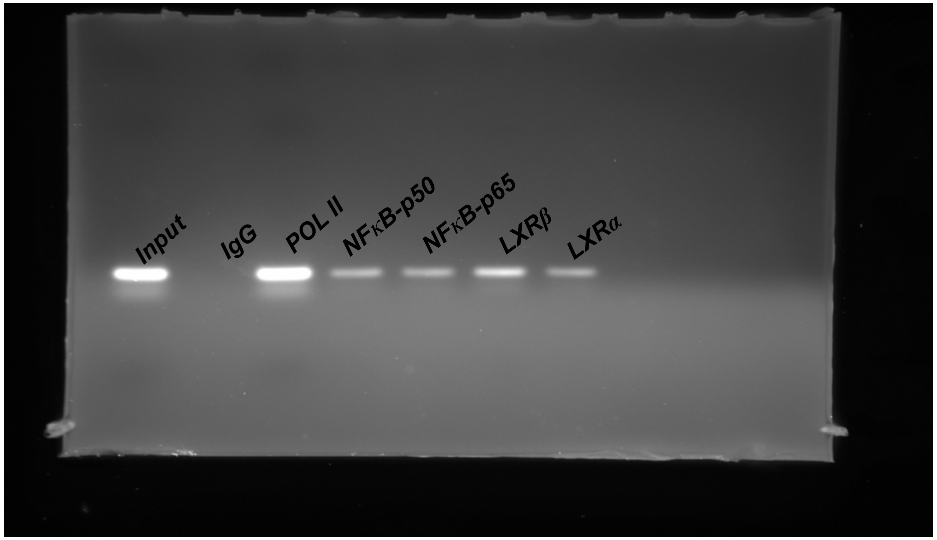

DSS/PN wt

Supplementary Figure5B-1-3

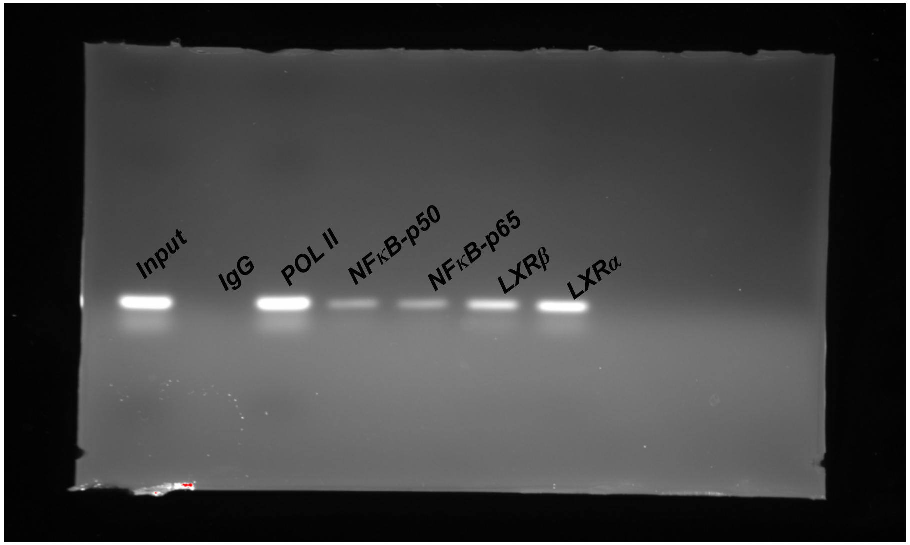

14day DSS/PN Il1r $^{-/-}$

Chromatin immuno precipitation (ChIP) on liver homogenate from WT chow mice, WT DSS/PN mice, and *Il1r*$^{-/-}$DSS/PN mice probing with specific antibodies for either LXRα,LXRβ, NFκB p50 and p65 subunits binding to the mouse Abcg5 promotor to LXRβE binding site. Represented as semi-quantitative data.

mAbcg5/8 NFκBE (intergenic region)

Supplementary Figure5B-2-1

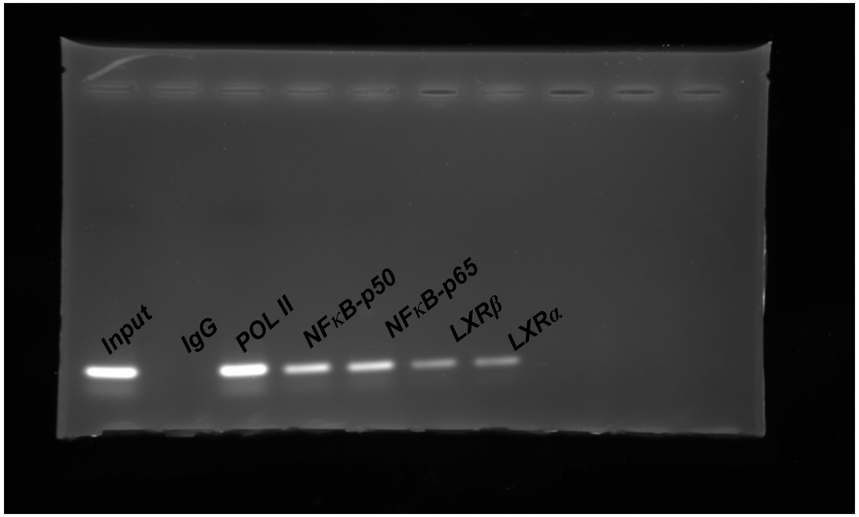

Chow

Supplementary Figure5B-2-2

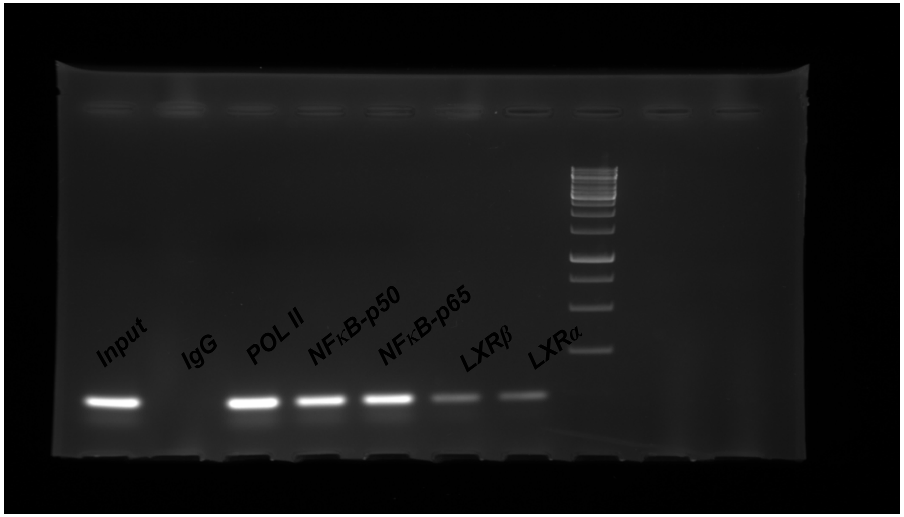

DSS/PN wt

Supplementary Figure5B-2-3

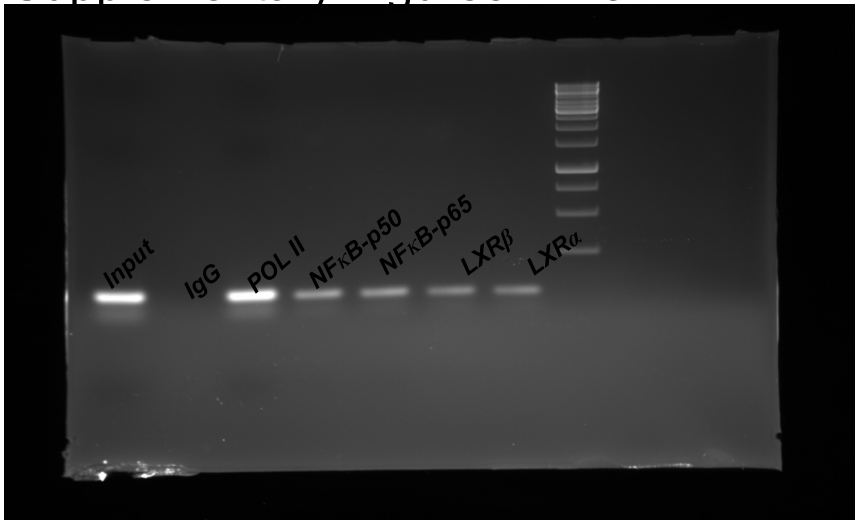

14day DSS/PN Il1r $^{-/-}$

Chromatin immuno precipitation (ChIP) on liver homogenate from WT chow mice, WT DSS/PN mice, and *Il1r*$^{-/-}$DSS/PN mice probing with specific antibodies for either LXRα,LXRβ , NFκB p50 and p65 subunits binding to the mouse Abcg5/G8(intergenic region)promotor to NFκBE binding site. Represented as semi-quantitative data.

Supplementary Figure 7

Uncropped gel images of Western and CHiP assays depicted in Figures 2, 4 and 5 of the main manuscript.
